# Bioactive Brominated Oxindole Alkaloids from the Red Sea Sponge *Callyspongia siphonella*

**DOI:** 10.3390/md17080465

**Published:** 2019-08-09

**Authors:** Seham S. El-Hawary, Ahmed M. Sayed, Rabab Mohammed, Hossam M. Hassan, Mostafa E. Rateb, Elham Amin, Tarek A. Mohammed, Mohamed El-Mesery, Abdullatif Bin Muhsinah, Abdulrhman Alsayari, Harald Wajant, Mohamed A. Anany, Usama Ramadan Abdelmohsen

**Affiliations:** 1 Department of Pharmacognosy, Faculty of Pharmacy, Cairo University, 11787 Cairo, Egypt; 2 Department of Pharmacognosy, Faculty of Pharmacy, Nahda University, 62513 Beni-Suef, Egypt; 3 Department of Pharmacognosy, Faculty of Pharmacy, Beni-Suef University, 62514 Beni-Suef, Egypt; 4 Marine Biodiscovery Centre, School of Natural and Computing Sciences, University of Aberdeen, Scotland AB24 3UE, UK; 5 School of Computing, Engineering and Physical Sciences, University of the West of Scotland, Paisley PA1 2BE, UK; 6 Marine Invertebrates, National Institute of Oceanography and Fisheries, Red Sea Branch, 84511 Hurghada, Egypt; 7 Division of Molecular Internal Medicine, Department of Internal Medicine II, University Hospital Würzburg, Grombühlstr. 12, 97080 Würzburg, Germany; 8 Department of Biochemistry, Faculty of Pharmacy, Mansoura University, 35516 Mansoura, Egypt; 9 Department of Pharmacognosy, College of Pharmacy, King Khalid University, Abha 61441, Saudi Arabia; 10 Division of Genetic Engineering and Biotechnology, Department of Microbial Biotechnology, National Research Centre, El Buhouth Street, Dokki, 12622 Giza, Egypt; 11 Department of Pharmacognosy, Faculty of Pharmacy, Minia University, 61519 Minia, Egypt

**Keywords:** *Callyspongia siphonella*, LC-HRESIMS, metabolomic profiling, oxindole alkaloids, tisindoline, antibacterial, antibiofilm, antitrypanosomal, anticancer

## Abstract

In the present study, LC-HRESIMS-assisted dereplication along with bioactivity-guided isolation led to targeting two brominated oxindole alkaloids (compounds **1** and **2**) which probably play a key role in the previously reported antibacterial, antibiofilm, and cytotoxicity of *Callyspongia siphonella* crude extracts. Both metabolites showed potent antibacterial activity against Gram-positive bacteria, *Staphylococcus aureus* (minimum inhibitory concentration (MIC) = 8 and 4 µg/mL) and *Bacillus subtilis* (MIC = 16 and 4 µg/mL), respectively. Furthermore, they displayed moderate biofilm inhibitory activity in *Pseudomonas* *aeruginosa* (49.32% and 41.76% inhibition, respectively), and moderate in vitro antitrypanosomal activity (13.47 and 10.27 µM, respectively). In addition, they revealed a strong cytotoxic effect toward different human cancer cell lines, supposedly through induction of necrosis. This study sheds light on the possible role of these metabolites (compounds **1** and **2**) in keeping fouling organisms away from the sponge outer surface, and the possible applications of these defensive molecules in the development of new anti-infective agents.

## 1. Introduction

Marine sponges are prolific producers of diverse bioactive secondary metabolites that were reported over the years [1,2]. These sessile marine animals developed a defensive chemical arsenal to keep small animals and plants (fouling organisms) away from settling on their outer surfaces [3]. Furthermore, these metabolites may also be used to regulate symbiotic bacterial populations known to associate with sponges [4]. The genus *Callyspongia* belongs to the order Haplosclerida, family Callyspongiidae. *Callyspongia siphonella* (Levi, 1965) is widely distributed throughout the Gulfs of Aqaba and Suez from shallow water, but mostly deeper than 5 m [5]. Several bioactive secondary metabolites including sipholane triterpenes [6], polyacetylenic alcohols [7], and amides [8] were isolated from different members of this genus. In addition to these anticancer metabolites, a number of steroidal anti-inflammatory compounds were also reported from this genus [9]. *Pseudomonas aeruginosa* biofilms can cause serious infections especially for the elderly and immunocompromised patients. Unfortunately, such infections often cannot be treated efficiently with common antibiotics [10,11]. A number of *Callyspongia* crude extracts showed potent antibacterial and antibiofilm activities toward several pathogenic bacterial strains, particularly *Pseudomonas aeruginosa* PA01 [12]. In the present study, bioactivity-guided fractionation together with LC-HRESIMS-assisted chemical investigation of the Red Sea sponge *C. siphonella* led to the isolation of two antibacterial brominated oxindole alkaloids (**1**,**2**) from a natural source for the first time along with several known metabolites. Compounds **1** and **2** showed considerable anti-biofilm activity in *P. aeruginosa* and moderate in vitro antitrypanosomal activity. Moreover, they induced strong cell death in HT29, OVCAR-3, and MM.1S cancer cell lines after 24-h stimulation.

## 2. Results and Discussion

### 2.1. Metabolomic Profiling

**Figure 1:**
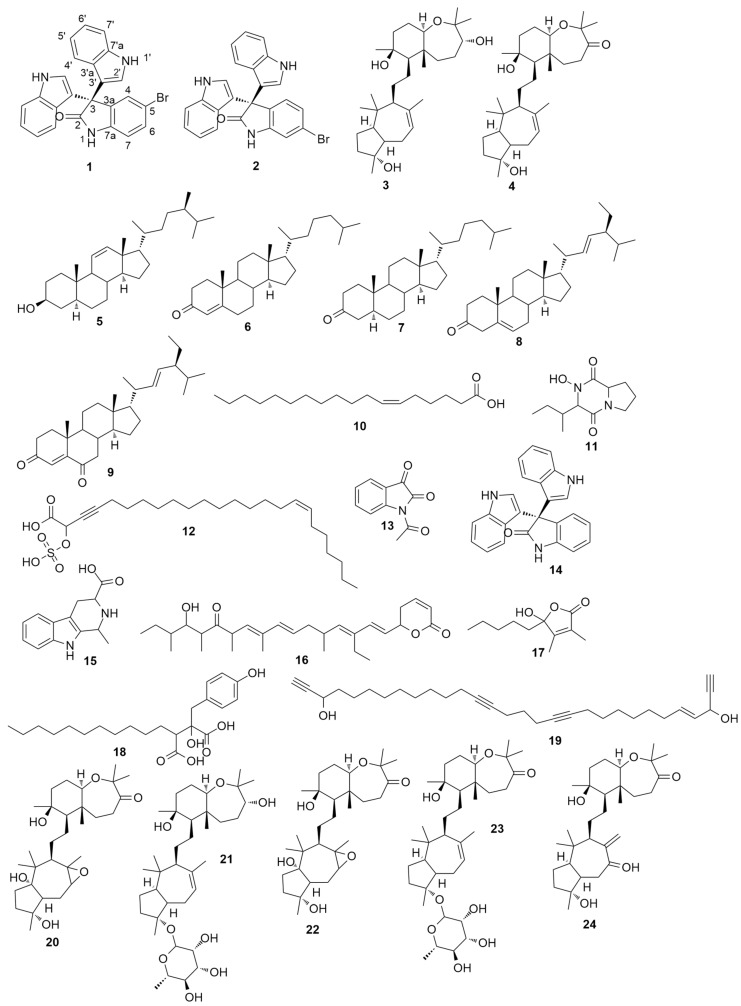
Identified compounds by dereplication with LC-HRESIMS.

To date, a growing number of secondary metabolites were identified from *Callyspongia siphonella* with diverse pharmacological effects [13]. Sipholane triterpenoids are considered the main characteristic metabolites of this sponge. This class of metabolites was found to show a potent reversal of multidrug resistance in tumor cells that overexpressed P-glycoprotein (P-gp) [14]. In addition, there are many polyactylenes, polyketides, and alkaloids that were also reported from *C. siphonella.* In view of that, metabolomic profiling of the marine sponge *C. siphonella* using LC-HRESIMS for dereplication purpose resulted in the characterization of a variety of metabolites (Figure 1 and Figure S1) of which sipholane triterpenes were found to be prevalent (Table 1). From the AntiMarine and Dictionary of Natural Products (DNP) databases, a total of 24 compounds were tentatively identified. Seven compounds out of these were members of the P-glycoprotein inhibitors, sipholane triterpenoids (**3**,**4**,**20–24**). Additionally, the antiproliferative metabolites, callystatin A (**16**), hydroxydihydrobovolide (**17**), callyspongidic acid (**18**), and callyspongendiol (**19**), were also dereplicated in the *n*-hexane fraction [15]. All sipholane triterpenes are presumably derived from the cyclization of triepoxysqualenes. Detection of several mass ion peaks proposed to be the biosynthetic intermediates of sipholane triterpenes supported the previously suggested biosynthetic pathway (Scheme S1) [6]. Similarly, compounds **11**, **12**, **13**, **14**, and **15** were detected in the ethyl acetate fraction. Moreover, intermediate compounds suggesting that they were involved in the biosynthesis of trisindoline (**14**) and its brominated derivatives (compounds **1** and **2**) from tryptophan [16,17] were also found (Scheme S2). Tisindoline (**14**) is an antibiotic indole trimer previously isolated from marine *Vibrio* and *Shewanella* species [18,19]. In addition to the identified hits (Figure 1), other unidentified molecular formulae were detected, particularly in the ethyl acetate fraction, suggesting the presence of further unknown metabolites. These findings, together with the primary antibacterial screening, prioritized the ethyl acetate fraction for a further chemical investigation to isolate the active metabolites. 

### 2.2. Structure Characterization of the Isolated Compounds

The ethanolic extract of *C. siphonella* was partitioned with different organic solvents with increasing polarity. The *n*-hexane and ethyl acetate fractions displayed weak to moderate antibacterial activities. Further bioactivity-guided fractionation of the ethyl acetate fraction, which showed several new hits upon LC-HRESIMS dereplication, led to the isolation of compounds **1** and **2**. 

**Figure 2:**
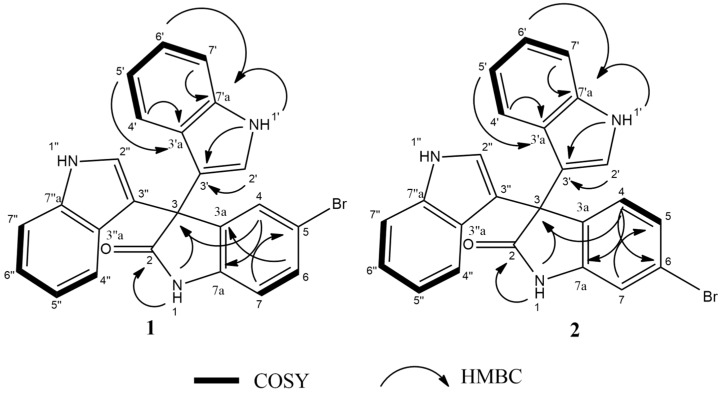
^1^H–^1^H COSY and key HMBC correlations of compounds **1**,**2**.

Compound **1** was isolated as buff powder; the molecular formula C_24_H_16_ON_3_Br was suggested on the basis of a positive HRESIMS ion at *m/z* 442.0554 [M + H]^+^, indicating 18 degrees of unsaturation. ^1^H-NMR spectral data of **1** (Table 2) in deuterated dimethyl sulfoxide (DMSO-*d_6_*) suggested the presence of two exchangeable protons (*δ*_H_ 10.77, 11.03) due to NH. In addition, the splitting pattern of resonances at *δ*_H_ 6.97 (H-7), 7.42 (H-6), and 7.30 (H-4), together with the ^1^H–^1^H COSY correlation between (H-6) and (H-7), suggested the presence of a 1,3,4-trisubstituted benzene ring. The DEPTQ spectrum (Table 2) displayed 16 signals, with 15 *sp^2^* aromatic carbons including eight CH groups and seven quaternary carbons, and one *sp^3^* aliphatic quaternary carbon (C-3). The spectra also revealed an aminocarbonyl at *δ*_C_178.9 (C-2). The assignment of protonated carbons was achieved by the ^1^H–^13^C HSQC data. The key HMBC correlations (Figure 2) from H-4 (*δ*_H_ 7.30, s, 1H) to C-3 (*δ*_C_ 53.2), and from the exchangeable proton H-1(*δ*_H_ 10.77) to the carbonyl carbons C-2 (*δ*_C_ 178.9) and C-3a (*δ*_C_ 137.44) together with the previous reported data [18], suggested the presence of a brominated indolin-2-one skeleton at C-5 (*δ*_C_ 114). The presence of two identical indole moieties linked to brominated oxindole moity at C-3 (*δ*_C_ 53.2) was figured out from the ^1^H-NMR spectral data (Table 2) and the ^1^H–^13^C HMBC correlations (Figure 2). According to these data, HRESIMS analysis, and previously published data [20], compound **1** was identified as 5-bromo trisindoline.

Compound **2** was isolated as a white amorphous powder. The molecular formula C_24_H_16_ON_3_Br was suggested on the basis of the positive HRESIMS ion at *m/z* 442.0554 [M + H]^+^, indicating 18 degrees of unsaturation. HRESIMS and ^1^H and ^13^C-NMR spectra of **2** (Table 2) were almost identical to that of compound **1**. In addition, the similar two-dimensional (2D)-NMR correlations indicate that compounds **1** and **2** are positional isomers, where the bromine is attached to C-6 instead of C-5. This different attachment was confirmed from the ^1^H–^13^C HMBC correlation between H-4 (*δ*_H_ 7.16, d, 8Hz, 1H) and C-2 (*δ*_C_ 179); hence, compound **2** was identified as 6-bromo trisindoline.

Both 5-bromotrisindoline (**1**) and 6-bromotrisindoline (**2**) are brominated indole trimers, previously reported as synthetic intermediates [20,21] and isolated herein for the first time from a natural source. Their parent non-halogenated trisindoline (**14**) was previously isolated from a marine bacterium and exhibited significant antibacterial activity [18]. Only 5-bromotrisindoline (**1**) was reported as an α-glycosidase enzyme inhibitor [20]. ^1^H-NMR and ^13^C-NMR assignments of 5-bromotrisindoline (**1**) and 6-bromotrisindoline (**2**) are reported here for the first time based on extensive HSQC, COSY, and HMBC analysis (Figures S3–S21, Supplementary Materials).

Further known metabolites, sipholenol A (**3**) and sipholenone A (**4**) [22], along with five steroidal compounds, callysterol (**5**) [9], cholestenone (**6**), 5α-cholestanone (**7**) [23], stigmasterone (**8**) [24], and stigmasta-4,22-dien-3,6-dione (**9**) [25], and one major fatty acid, petroselenic acid (**10**) [26,27], were also isolated from the *n*-hexane fraction of *C. siphonella* (Figure 1). All isolated known metabolites were identified based on their accurate mass analyses and comparison of their NMR spectroscopic data with those reported in the literature (Figures S22–S47, Supplementary Materials). The triterpenes sipholenol A (**3**) and sipholenone A (**4**) are major metabolites in *C. siphonella*. Sipholenol A (**3**) was previously reported as a potent P-glycoprotein inhibitor in multidrug-resistant cancer cells [14]. Callysterol (**5**) is a characteristic sterol in *C. siphonella* which exhibited in vitro anti-inflammatory activity [9]. The steroidal compounds (**6–9**), along with the fatty acid petroselenic acid (**10**), were isolated here from *C. siphonella* for the first time.

### 2.3. Antibacterial Activity

For primary antibacterial screening, the crude ethanolic extracts, all fractions, and the isolated compounds were evaluated against *Staphylococcus aureus* (ATCC 25923), *Bacillus subtilis* (ATCC 5230), *Escherichia coli* (ATCC 25922), and *Pseudomonas aeruginosa* (ATCC 9027). The antibacterial activity was recorded as an inhibition zone diameter and measured as “mm” (Table 3). Ampicillin and gentamicin were used as a positive control. The ethyl acetate fraction showed the highest inhibition against *Staphylococcus aureus* (6.6 mm), *Bacillus subtilis* (5.4 mm), and *Escherichia coli* (1.5 mm), while the *n*-hexane fraction exhibited mild antibacterial activity with inhibition zones ranging from 1 to 2 mm. These findings are in great accordance with the previously reported antibacterial activity of *C. siphonella* extracts [28]. Compounds **1** and **2** derived from the ethyl acetate fraction displayed potent antibacterial activity against Gram-positive bacteria *S. aureus* and *B. subtilis* with inhibition zones of 17.5 mm and 18 mm, and 15 mm and 16.4 mm, respectively (Table 4). With respect to Gram-negative bacteria *E. coli* and *P. aeruginosa*, **1** and **2** showed weak antibacterial activity with inhibition zones of 0.5 mm and 1.3 mm, and 0.2 mm and 1.1 mm, respectively. The other isolated compounds (**3–10**) did not exhibit any inhibition against the tested bacterial strains. The minimum inhibitory concentration (MIC) values of **1** and **2** that showed antibiotic activity in disc diffusion tests were determined (Table 5). It was observed that the MIC value of **2** against *S. aureus* (16 µg/mL) was higher than that of **1** (8 µg/mL). However, both compounds exhibited potent inhibitory effect toward *B. subtilis* with an MIC value of 4 µg/mL. In a previous study [18], their parent compound, trisindoline (**14**), showed antibacterial activity against both Gram-positive and Gram-negative bacterial strains; hence, incorporation of a bromine atom into the oxindole moiety of trisindoline (**14**) reduces the antibacterial spectrum of these molecules. From the previous observations, we can conclude that the bromotrisindolines (**1,2**) may be the main metabolites responsible for the antibacterial activity of *C. siphonella* extract against Gram-positive bacteria. 

### 2.4. Antibiofilm Activity

As demonstrated from the antibacterial screening, metabolites **1** and **2** did not exhibit any inhibition against the Gram-negative bacteria, *P. aeruginosa* PAO1. However, compounds that have no direct growth inhibitory effect on the pathogenic organisms still have the potential to act as biofilm formation inhibitors. As outlined in a recent review [29], many categories of natural products, including marine invertebrate-derived metabolites can stop the formation of biofilms and, thus, comprise candidates for adjuvant therapy as boosters of the common antibiotics. Therefore, all isolated compounds were tested against *P. aeruginosa* to determine if they could inhibit biofilm formation. Compound **1** reduced biofilm formation slightly more (49.32% inhibition) than **2** (41.76%) at concentrations of 128 µg/mL (0.5 MIC) (Table 6). The remaining compounds were inactive at tested concentrations. To the best of our knowledge, none of the derivatives of trisindoline antibiotic are so far associated with inhibition of biofilm formation in human pathogens. Therefore, further mechanistic studies on these compounds are needed.

### 2.5. Antitrypanosomal Activity

African trypanosomiasis, which is also known as sleeping sickness, is a fatal and neglected disease caused by *Trypanosoma brucei*, affecting mostly the poor people living in tropical and subtropical zones [30]. The increasing resistance to the current medications necessitated the development of novel alternatives to fight this disease. Natural products will continue to play a vital role in the search for new antitrypanosomal drugs. All isolated compounds were tested against the sleeping sickness parasite, *T. brucei*. Both metabolites **1** and **2** demonstrated moderate antitrypanosomal activity after 48 and 72 h with half maximal inhibitory concentration (IC_50_) values of 15.36 (48 h) and 13.47 (72 h), and 12.35 (48 h), and 10.27 (72 h) µM, respectively. However, the other isolated compounds were found inactive against *T. brucei*. 

### 2.6. Cytotoxic Activity

**Figure 3:**
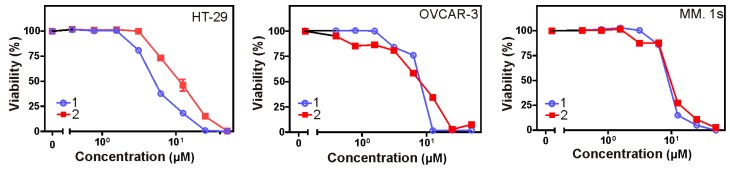
Cytotoxic effects of compounds **1** and **2** on human colon cancer (HT-29), human ovarian cancer (OVACR-3), and multiple myeloma (MM.1s) cell lines. The cells were stimulated overnight with increasing concentrations of compounds **1** and **2**. on the next day, cellular viability was determined by crystal violet staining for HT-29 and OVACR-3, and 3-(4,5-dimethylthiazol-2-yl)-2,5-diphenyltetrazolium bromide (MTT) assay for MM.1s.

**Figure 4:**
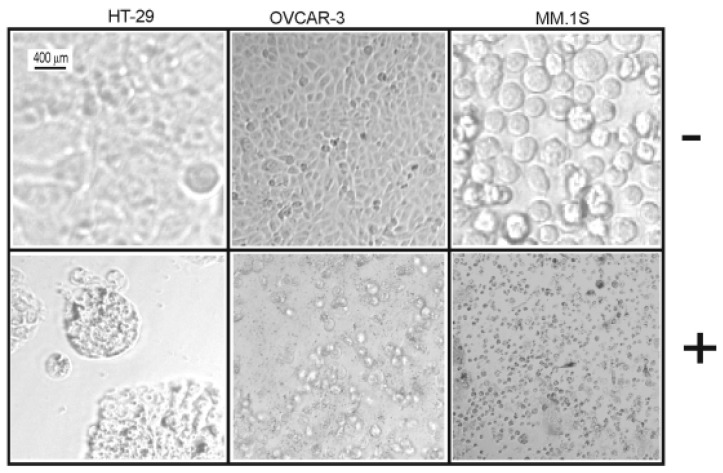
Morphological changes of HT-29, OVCAR-3, and MM.1S cells incubated overnight with compound (**1**) (20 µM) in comparison to untreated cells using an EVOS FL digital microscope.

**Figure 5:**
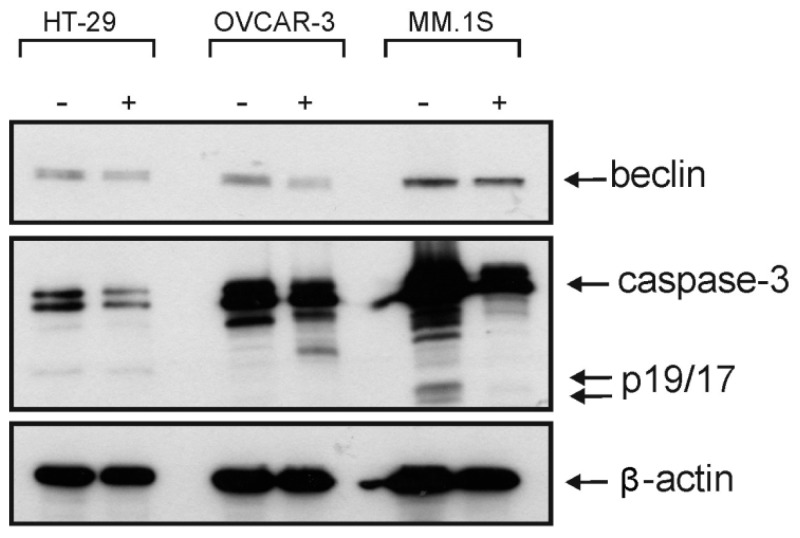
Western blotting analysis of **1** against beclin and caspase 3, the key proteins of autophagy and apoptosis, respectively.

Crystal violet and 3-(4,5-dimethylthiazol-2-yl)-2,5-diphenyltetrazolium bromide (MTT) assays were used to assess the cytotoxicity of all isolated metabolites against three different cancer cell lines over a concentration range of 0.01–50 µM. Both **1** and **2** revealed significant cytotoxic effects (Table 7) against all cell lines under investigation (human colon cancer cell line: HT-29, human ovarian cancer cell line: OVCAR-3, and multiple myeloma cell line: MM.1S). Furthermore, the microscopic pictures showed clear changes in cell morphology after overnight treatment with **1** (Figure 3 and Figure 4). However, the Western blotting analysis showed only a decrease in the level of procaspase-3 but unfortunately, we neither detected any split caspase-3 products, the key protein of apoptosis, nor changes in the level of beclin protein, one of the master proteins of autophagy (Figure 5). Moreover, **1** was not able to induce the upregulation of proinflammatory cytokines such as interleukin-8 (IL8) in MM.1S and HT-29 (Figure S2, Supplementary Materials). Our previous results and many previous publications showed that the programmed cell death is usually accompanied by the induction of proinflammatory signals such as IL8 [31,32]. This confirms the Western blotting results that compound **1** kills the cancer cells by non-programmed cell necrosis but not by programmed cell death mechanisms such as necroptosis, apoptosis, or autophagy. 

## 3. Material and Methods

### 3.1. Extraction and Fractionation

The marine sponge *C. siphonella* (2 kg) was collected in November 2015 from Hurghada along the Red Sea Coast (27°15048” north (N), 33°4903” east (E)) at depth of 7 m. A voucher sample (NIOF204/2015) was reserved at the Invertebrates Department, National Institute of Oceanography and Fisheries, Red Sea Branch, Hurghada, Egypt. Subsequently, sponge material was cut into small pieces, and then subjected to ultrasonic-assisted extraction with ethanol (3 × 500 mL). The resulted liquid extract was concentrated using a rotary evaporator (IKA^®^, Staufen, Germany). The obtained concentrated extract was suspended in distilled water and fractionated with *n*-hexane (4 × 200 mL), ethyl acetate (3 × 200 mL), and *n*-butanol (3 × 200 mL). All fractions were separately concentrated under reduced pressure and screened for their antibacterial activities.

### 3.2. Metabolomics Analysis

Metabolomic profiling was performed on the crude extract of *C. siphonella* on an Acquity Ultra Performance Liquid Chromatography system coupled to a Synapt G2 HDMS quadrupole time-of-flight hybrid mass spectrometer (Waters, Milford, CT, USA). Chromatographic separation was carried out on a BEH C18 column (2.1 × 100 mm, 1.7 μm particle size; Waters, Milford, CT, USA) with a guard column (2.1 × 5 mm, 1.7 μm particle size) and a linear binary solvent gradient of 0–100% eluent B over 6 min at a flow rate of 0.3 mL∙min^−1^, using 0.1% formic acid in water (*v*/*v*) as solvent A and acetonitrile as solvent B. The injection volume was 2 μL and the column temperature was 40 °C. Ms converter software was used in order to convert the raw data into divided positive and negative ionization files. Obtained files were then subjected to the data mining software MZmine 2.10 (Okinawa Institute of Science and Technology Graduate University, Japan) for deconvolution, peak picking, alignment, deisotoping, and formula prediction. The databases used for the identification of compounds were: MarinLit: http://pubs.rsc.org/marinlit/, and Dictionary of Natural Products (DNP) 2018: http://dnp.chemnetbase.com/faces/chemical/ChemicalSearch.xhtml. 

### 3.3. Assessment of Antibacterial Activity

The tested microorganisms used in this study included Gram-positive (*Staphylococcus aureus*, ATCC 25923 and *Bacillus subtilis* ATCC 5230) and Gram-negative (*Escherichia coli* ATCC 25922 and *Pseudomonas aeruginosa* ATCC 9027) pathogenic bacteria. An agar well diffusion assay was used to investigate the antibacterial activity of all sponge extracts and fractions [33]. Minimal inhibitory concentrations (MIC) were determined by the broth dilution method for isolated compounds according to Clinical and Laboratory Standards Institute (CLSI) protocol [34].

### 3.4. Bioactivity-Guided Isolation of the Major Metabolites

After a preliminary assessment of all prepared fractions and extracts for antibacterial activities, the ethyl acetate fraction (2 g) was the most active against Gram-positive bacteria. Further fractionation on silica gel column (60–120 mesh, Fluka^®^) using *n-*hexane/ethyl acetate in a gradient elution, resulted in three sub-fractions (codes **F1–F3**). Afterward, the active sub-fraction **F3** (400 mg) was chromatographed on Agilent^®^ 1260 Infinity preparative HPLC using acetonitrile in water (70–100%) for 20 min followed by 100% acetonitrile for the next 10 min at a 20 mL/min flow rate to afford **1** (21 mg) and **2** (25 mg). Similarly, the less active *n*-hexane fraction was fractionated on a silica gel column using *n-*hexane/ethyl acetate in a gradient elution to give six sub-fractions (codes **F1–F6**). Subfraction **F1** (600 mg) was chromatographed on a silica gel column using *n-*hexane/ethyl acetate in a gradient elution to afford **10** (40 mg) and **7** (15 mg). In the same manner, subfraction **F2** (350 mg) was chromatographed on silica gel column using *n-*hexane/ethyl acetate in a gradient elution to afford **6** (15 mg) and **8** (20 mg). Furthermore, subfractions **F3–F5** (250 mg) were combined and chromatographed on a silica gel column using dichloromethane/methanol in a gradient elution to afford **9** (15 mg), **5** (30 mg), **4** (50 mg), and **3** (100 mg). The structure of the isolated compounds was elucidated by comparison of their spectral data (MS and NMR) with those reported in the literature. MS and NMR spectra were recorded on Agilent series 1100 SL (Agilent CO, Santa Clara, CA, USA) and Bruker Avance III 400 MHz (Bruker AG, Switzerland), respectively.

### 3.5. Screening of Antibiofilm Activity

The assay was performed as previously described by Drenkard and Ausubel (2002) [35] with minor modifications. Briefly, *Pseudomonasaeruginosa*PAO1 was cultured in lysogeny broth (LB) medium (Himedia^®^, Mumbai, India). After incubation, cultures were vortexed, diluted 1:100 in fresh LB medium for biofilm formation, and cultured with or without the tested natural products and azithromycin (AZM) (Sigma Aldrich^®^, Munich, Germany) for 24 h at 37 °C. Then, the non-adherent cells were washed with phosphate-buffered saline (PBS), while adherent ones were subjected to 1% crystal violet staining for 15 min. The wells were washed again with water to get rid of excess stain. After the wells dried, the crystal violet obligated to the biofilm was extracted with 95% ethanol. The crystal violet solution absorbance (in optical density units, OD) was measured at 570 nm. AZM was reported as a biofilm inhibitor at the sub-inhibitory concentration [36]; thus, it was used as a positive control in the bioassay. Percentage inhibition was calculated according to the following rule: 

$$\%\;\mathrm{of}\;\mathrm{inhibition}\;=\;\frac{\mathrm{Control}\;\mathrm{OD}\;-\;\mathrm{Test}\;\mathrm{OD}}{\mathrm{Control}\;\mathrm{OD}}\;\times 100.$$

### 3.6. Antitrypanosomal Activity Testing

The antitrypanosomal activity was tested against *Trypanosoma brucei* strain TC221 according to the Huber and Koella protocol [37]. The growth inhibitory activity was measured using an MR 700 microplate reader at wavelengths of 550 and 650 nm. IC_50_ values were calculated by linear interpolation of three independent measurements. Samples were considered active when their IC_50_ values were less than 20 μM.

### 3.7. Cytotoxic Activity Assessment

Cell lines were maintained in earth RPMI 1640 medium (HT-29 and MM.1S cells) or in DMEM medium (OVCAR-3 cells) containing 10% heat-inactivated fetal bovine serum (FBS), and were grown at 37 °C and 5% CO_2_. Anti-caspase-3 (#9662) and anti-beclin (#3495S) were purchased from Cell Signaling (Beverly, MA, USA), and anti-β-actin (monoclonal AC015) from Sigma (Louis, Mo 363103 USA). Cell lines were counted and seeded on 96-well cell culture plates (2 × 10^4^ cells/well in the case of HT-29 and OVCAR-3 and 6 × 10^4^ cells/well in the case of MM.1S cells). Cells were stimulated either in the next day in the case of HT-29 and OVCAR-3 or on the same day in the case of MM.1S cells with the indicated concentrations of each compound overnight in triplicates. Cell viability was assessed either by crystal violet staining in the case of HT-29 and OVCAR-3 or by 3-(4,5-dimethylthiazol-2-yl)-2,5-diphenyltetrazolium bromide (MTT) assay in the case of MM.1S cells [38]. To normalize cell viability values, each plate included a triplicate of untreated cells considered as 100% viability, and a triplicate of cells treated with a cytotoxic mixture (200 ng/mL TNF, 200 ng/mL CD95L, 200 ng/mL TRAIL, 5 μg/mL CHX, 1% (*w*/*v*) sodium azide 20%) considered as 0% viability. All other viability values were normalized according to the averages of these triplicates and analyzed by the Graph Pad Prism 5 software (La Jolla, CA, USA).

### 3.8. Western Blotting

Cells were seeded in six-well plates (1 × 10^6^/well) and stimulated with 40 µM of compound **4**. In the next day, cells were harvested in ice-cold PBS and washed by centrifuging cells twice in PBS and centrifuged for 4 min at 1200 rpm (4°C). Then, total cell lysates were prepared as described before in our previous work [32]. The membranes were incubated overnight with primary antibodies and antigen-antibody reactions were performed in the next day using the corresponding secondary antibodies (anti-mouse-HRP, Dako-Cytomation, Hamburg, Germany; anti-rabbit-HRP, Cell Signaling Technology). The specific bands were visualized using ECL detection reagents for Western blotting (Amersham Biosciences, Germany).

### 3.9. IL8 ELISA

Firstly, 2 × 10^4^ of MM.1S and HT-29 cells per well were seeded in 96-well plates. On the next day, the medium was changed to reduce the background of constitutive IL8 production, and cells were incubated with the indicated reagent(s) for 16 h. The supernatants were collected and analyzed for production of IL8 using the human IL8 ELISA (enzyme-linked immunosorbent assay) kit BD Biosciences (Heidelberg, Germany) according to the instructions of the supplier.

## 4. Conclusion

The present study clearly highlighted the efficacy of LC–MS profiling when coupled to bio-guided drug discovery from marine invertebrates to speed up the traditionally long processes of identifying an active metabolite by consecutive isolation from crude extracts. Dereplication studies based on the chemotaxonomic sorting helped in the identification of putative active metabolites, whereas structural assignment of the isolated compounds, employing both HR-MS and NMR, confirmed the identified hits. Thus, bio-guided isolation coupled to LC–MS metabolomic profiling of the Red Sea sponge *C. siphonella* led to the characterization of two brominated indole alkaloids (**1,2**), which were not isolated from natural sources before. Moreover, metabolomics analysis gave insight into the metabolic pathways required for the biosynthesis of compounds **1** and **2** from tryptophan, together with the characteristic sipholane triterpenoids from squalene. Compounds **1** and **2** showed excellent in vitro antibacterial activity against the tested Gram-positive strains and displayed significant anti-biofilm activity in *P. aeruginosa*. Moreover, they exhibited considerable in vitro antitrypanosomal activity, and were able to trigger potent cell death in a few tumor cell lines. Western blotting together with IL8 assay results suggested that compounds **1** and **2** could mediate their cytotoxicity via necrosis induction. The present investigation sheds the light on how *C. siphonella* can defend itself chemically within its hostile marine environment and the possible utilization of its defensive metabolites in the future development of new anti-infective agents. Further in vivo biological evaluation of these interesting bis-indoles will be of value for future drug development.

## Figures and Tables

**Table 1:** Dereplication of the metabolites previously identified from *Callyspongia siphonella.*

NP	Compound	RT (min)	[M + H]^+^	Calculated Mass	Chemical Formula
**1**	Compound **1** *^a^*	3.21	442.0552	442.0555	C_24_H_17_N_3_OBr
**2**	Compound **2** *^a^*	3.32	442.552	442.0555	C_24_H_17_N_3_OBr
**3**	Sipholenol A *^c^*	5.96	477.3941	477.3944	C_30_H_53_O_4_
**4**	Sipholenone A *^c^*	6.41	475.3785	475.3787	C_30_H_51_O_4_
**5**	Callysterol *^c^*	7.12	401.381	401.3783	C_28_H_49_O
**6**	Cholestenone *^b^*	7.38	385.3472	385.347	C_27_H_45_O
**7**	5α-cholestanone *^b^*	7.41	387.3625	387.3627	C_27_H_47_O
**8**	Stigmasterone *^b^*	7.49	411.3634	411.3637	C_29_H_47_O
**9**	stigmasta-4,22-dien-3,6-dione *^b^*	6.95	425.345	425.342	C_29_H_45_O_2_
**10**	Petroselenic acid *^b^*	8.91	283.2634	283.2637	C_18_H_35_O_2_
**11**	Callyspongidipeptide A	2.26	227.139	227.1396	C_11_H_19_N_2_O_3_
**12**	Callysponginol sulfate A	2.6	459.2783	459.278	C_24_H_43_O_6_S
**13**	*N*-acetyl isatin *^b^*	2.7	190.0501	190.0504	C_10_H_8_NO_3_
**14**	Trisindoline *^b^*	2.86	364.1456	364.145	C_24_H_18_N_3_O
**15**	1,2,3,4-tetrahydro-1- methyl-β-carboline-3-carboxylic acid	2.99	231.1133	231.1134	C_13_H_15_N_2_O_2_
**16**	Callystatin A	3.83	457.3325	457.3318	C_29_H_45_O_4_
**17**	Hydroxydihydrobovolide	3.88	199.1339	199.1334	C_11_H_19_O_3_
**18**	Callyspongidic acid	3.95	395.2429	395.2434	C_22_H_35_O_6_
**19**	Callyspongendiol	4.51	437.3413	437.3420	C_30_H_45_O_2_
**20**	15,16-epoxy-22-hydroxysipholen-one A	5.18	507.3676	507.3686	C_30_H_51_O_6_
**21**	Sipholenoside B	5.34	623.452	623.4523	C_36_H_63_O_8_
**22**	Sipholenol G	5.39	493.3889	493.3893	C_30_H_53_O_5_
**23**	Sipholenoside A	5.63	621.4368	621.4366	C_36_H_61_O_8_
**24**	Sipholenone C	6.26	489.3562	489.3580	C_30_H_49_O_5_

*^a^* Isolated in the present study as a new natural product. *^b^* Isolated in the present study for the first time from *C. siphonella*. *^c^* Previously reported from *C. siphonella* and confirmed by isolation.

**Table 2:** ^1^H- (400 MHz) and ^13^C-NMR (100 MHz) for compounds **1** and **2** in DMSO-*d6.*

Compound 1			Compound 2	
Position	*δ*_H,_ mult. (*J* in Hz)	*δ*_C__,_ Type	*δ*_H,_ mult. (*J* in Hz)	*δ*_C__,_ Type
**1-NH**	10.77 (br s, 1H)	-	10.75, (br s, 1H)	-
**2**	-	178.9, C	-	179, C
**3**	-	53.2, C	-	52.7, C
**3a**	-	137.4, C	-	134.3, C
**4**	7.30, (s, 1H)	127.8, CH	7.16, (d, 8Hz, 1H)	127.1, CH
**5**	-	114, C	7.12, (dd, 2Hz, 8Hz, 1H)	124.6, CH
**6**	7.42, (d, 8Hz, 1H)	131.2, CH	-	120.7, C
**7**	6.97, (d, 8Hz, 1H)	112.2, CH	7.14, (s, 1H)	112.8, CH
**7a**	-	141.1, C	-	143.5, C
**1-NH’, 1-NH’’**	11.03 (br s, 2H)	-	11.0, (br s, 1H)	-
**2′, 2′’**	6.89, (s, 2H)	124.9, CH	6.85, (d, 2.5Hz, 2H)	124.8, CH
**3′, 3′’**	-	113.6, C	-	114.0, C
**3′a, 3′’a**	-	126, C	-	126, C
**4′, 4′’**	7.22, (d, 8Hz, 2H)	121, CH	7.2, (d, 8Hz, 2H)	121, CH
**5′, 5′’**	6.83, (t, 8Hz, 2H)	119, CH	6.81, (t, 8Hz, 2H)	118.8, CH
**6′, 6′’**	7.04, (t, 8Hz, 2H)	121.5, CH	7.03, (t, 8Hz, 2H)	121.5, CH
**7′, 7′’**	7.39, (d, 8Hz, 2H)	112.2, CH	7.35, (d, 8Hz, 2H)	112.1, CH
**7′a, 7′’a**	-	137.4, C	-	137.4, C

**Table 3:** Inhibition zone diameter (mm) of the ethanol extract and fractions of *C. siphonella* on different bacterial strains (mean ± standard error (SE).

Tested Extract	*Staphylococcus aureus*	*Bacillus subtilis*	*Escherichia coli*	*Pseudomonas aeruginosa*
EtOH Ext	1.1 ± 0.5	1.2 ± 0.2	-	-
Hex	2.3 ± 0.9	1.1 ± 0.4	-	1 ± 0.4
EtOAc	6.6 ± 0.2	5.4 ± 0.3	1.5 ± 0.7	-
ButOH	-	0.5 ± 0.2	-	-
Ampicillin	13.7 ± 0.9	12.3 ± 1.2	3.9 ± 0.9	3.6 ± 0.3
Gentamicin	9.8± 1.2	10.1 ± 1.1	15.5 ± 0.1	14.8 ± 1.3

Ampicillin, gentamicin, extracts, and fractions (20 µg/mL DMSO). EtOH, ethanol extract; Hex, *n*-hexane fraction; EtOAc, ethyl acetate fraction; ButOH, *n*-butanol fraction. (-) indicates no growth inhibition found.

**Table 4:** Inhibition zone diameter (mm) of **1** and **2** on different bacterial strains (mean ± SE).

Tested compound	*S. aureus*	*B. subtilis*	*E. coli*	*P. aeruginosa*
**1**	17.5 ± 0.8	18 ± 0.1	0.5 ± 0.1	1.3 ± 0.3
**2**	15 ± 1.1	16.4 ± 0.9	0.2 ± 0.2	1.1 ± 0.7
Amikacin	23.5 ± 0.8	20.2 ± 0.6	15.4 ± 0.4	16.3 ± 0.9

**Table 5:** Minimal inhibitory concentration (MIC) (µg/mL) values of **1** and **2**.

Fungal metabolite	*S. aureus*	*B. subtilis*	*E. coli*	*P. aeruginosa*
**1**	8	4	>256	256
**2**	16	4	>256	256
Ampicillin	2	2	4	8
Gentamicin	16	8	0.5	1

**Table 6:** Antibiofilm activity of **1** and **2** in *Pseudomonas aeruginosa* PA01.

Isolated compound	% Inhibition
**1**	49.32 ± 1.18
**2**	41.76 ± 1.33
Azithromycin	52.62 ±1.23

Each compound was tested at concentration of 128 µg/mL (0.5 MIC). Azithromycin was tested at a concentration of 16 µg/mL (0.5 MIC).

**Table 7:** Cytotoxic effects of **1** and **2** expressed by half maximal inhibitory concentration (IC_50_) values.

Compounds	IC_50_ (µM) ^a^		
	HT-29	OVCAR-3	MM. 1S
**1**	8 ± 0.8	7 ± 0.3	9 ± 0.7
**2**	12.5 ± 0.3	9 ± 0.6	11 ± 0.9

^a^ Values are means of three independent experiments. human colon cancer cell line: HT-29; human ovarian cancer cell line: OVCAR-3; multiple myeloma cell line: MM.1S.

## Supplementary Materials

The following are available online at https://www.mdpi.com/1660-3397/17/8/465/s1: Schemes S1 and S2: Proposed metabolic pathways for sipholane triterpenoids and alkaloids in *Callyspongia siphonilla*; Figures S1–S47: One-dimensional and two-dimensional (1D and 2D) NMR spectra of isolated compounds.
