# Supplementary material for: Bioactive Brominated Oxindole Alkaloids from the Red Sea Sponge Callyspongia siphonella
Source: Mar Drugs. 2019 Aug 9;17(8):465. doi: 10.3390/md17080465 (PMC6723499; doi:10.3390/md17080465)
Supplement: Supplementary file 1 [file marinedrugs-17-00465-s001.pdf]

Supplementary Material

Bioactive Brominated Oxindole Alkaloids from the Red Sea Sponge *Callyspongia siphonella*

Seham S. El-Hawary [1], Ahmed M. Sayed [2], Rabab Mohammed [3], Hossam M. Hassan [3], Mostafa E. Rateb [3,4,5], Elham Amin [3], Tarek A. Mohammed [6], Mohamed El-Mesery [7,8], Abdullatif Bin Muhsinah [9], Abdulrhman Alsayari [9], Harald Wajant [7], Mohamed A. Anany [7,10,*] and Usama Ramadan Abdelmohsen [11,*].

[1] Department of Pharmacognosy, Faculty of Pharmacy, Cairo University, 11787 Cairo, Egypt,
[2] Department of Pharmacognosy, Faculty of Pharmacy, Nahda University, 62513 Beni-Suef, Egypt,
[3] Department of Pharmacognosy, Faculty of Pharmacy, Beni-Suef University, 62514 Beni-Suef, Egypt,
[4] Marine Biodiscovery Centre, School of Natural and Computing Sciences, University of Aberdeen, Scotland AB24 3UE, UK,
[5] School of Computing, Engineering and Physical Sciences, University of the West of Scotland, Paisley PA1 2BE, UK,
[6] Marine Invertebrates, National Institute of Oceanography and Fisheries, Red Sea Branch, 84511 Hurghada, Egypt,
[7] Division of Molecular Internal Medicine, Department of Internal Medicine II, University Hospital Würzburg, Grombühlstr. 12, 97080, Würzburg, Germany,
[8] Department of Biochemistry, Faculty of Pharmacy, Mansoura University, 35516 Mansoura, Egypt,
[9] Department of Pharmacognosy, College of Pharmacy, King Khalid University, Abha 61441, Saudi Arabia
[10] Division of Genetic Engineering and Biotechnology, Department of Microbial Biotechnology, National Research Centre, El Buhouth Street, Dokki, 12622 Giza, Egypt
[11] Department of Pharmacognosy, Faculty of Pharmacy, Minia University, 61519 Minia, Egypt
* Correspondence: mohamed_M@klinik.uni-wuerzburg.de (M.A.A.); usama.ramadan@mu.edu.eg (U. R.A.); Tel.: +49 931 201-71010 (M.A.A.); 2-86-2347759 (U.R.A.); Fax: +2-86-2369075 (U. R.A.).

Scheme S1. Proposed biogenesis of sipholane triterpenes in *C. siphonella* originating from squalane.

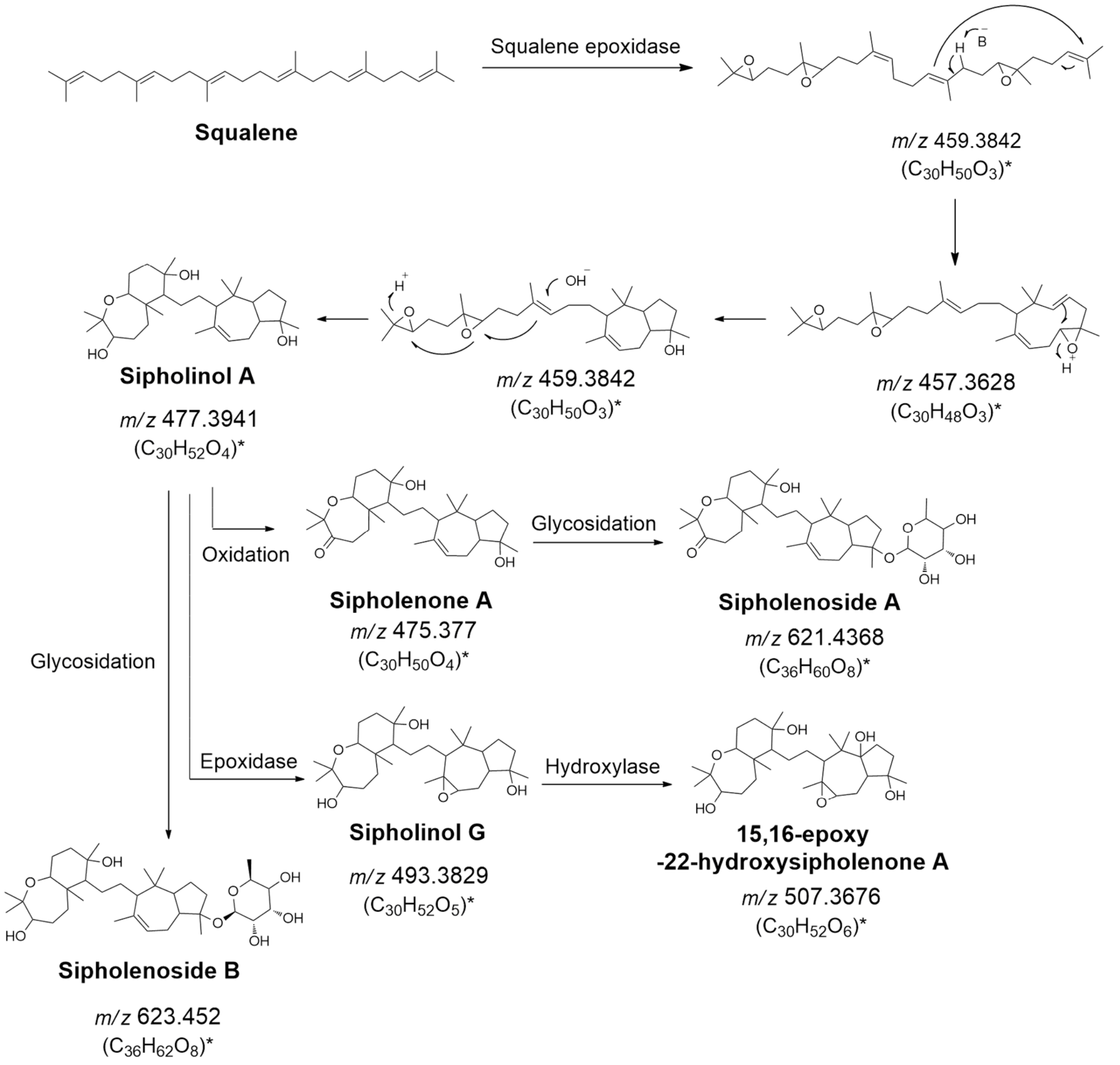

* indicate metabolites identified in the organic extracts of *C. siphonella*.

Scheme S2. Proposed biogenesis of indole alkaloids in *C. siphonella* originating from tryptophan.

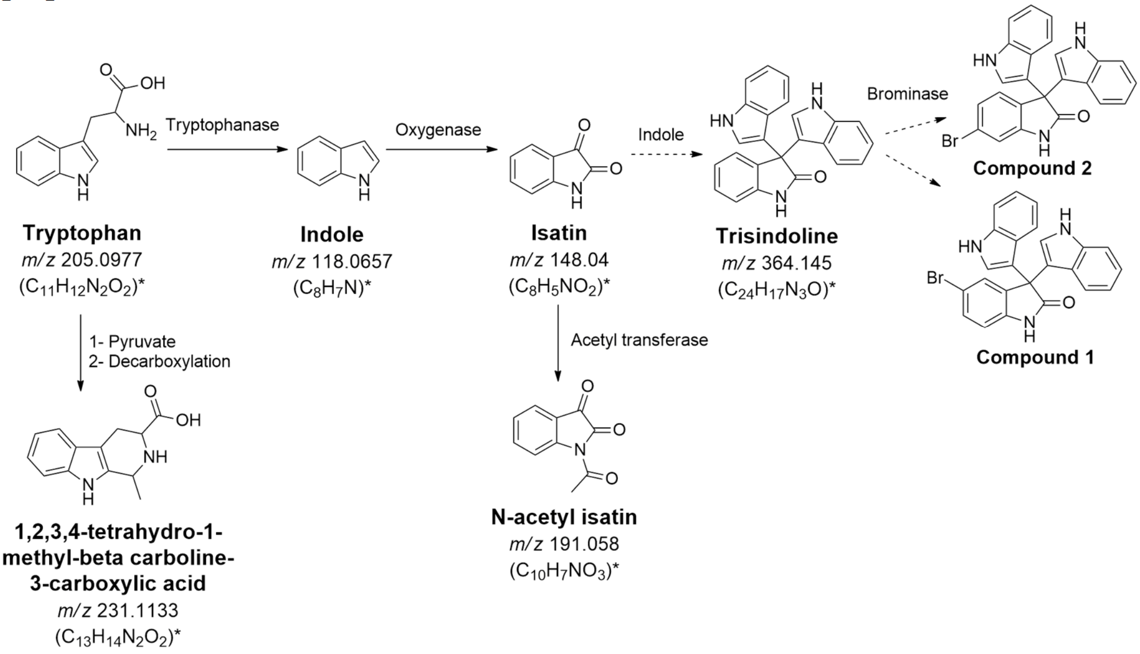

* indicate metabolites identified in the organic extracts of *C. siphonella*.

Arrows with solid line indicate pathway confirmed by enzyme assays (Found in the Kyoto Encyclopedia of Genes and Genomes (KEGG), arrows with dashed line indicate pathway proposed based on previous literatures.

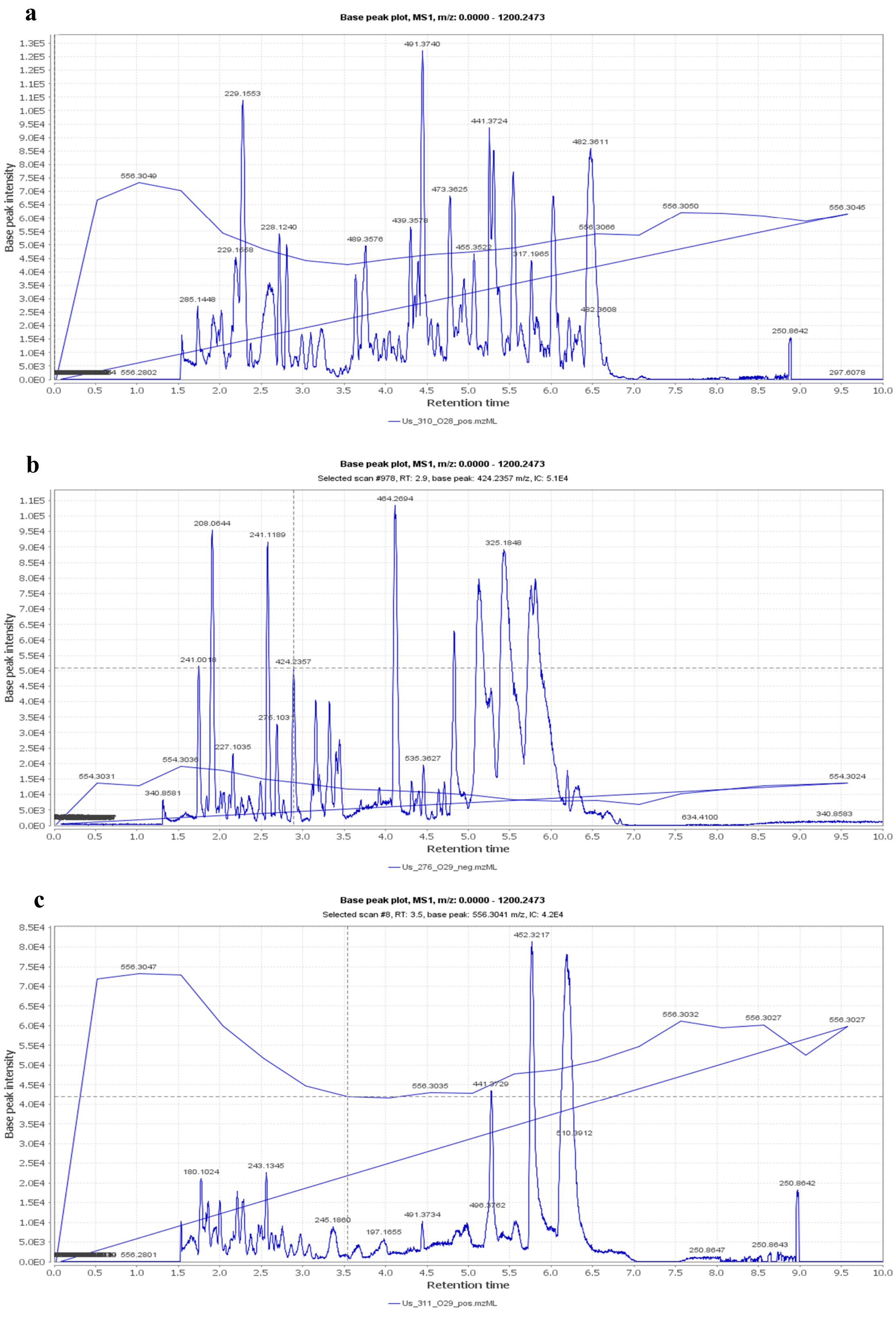

Fig.S1. Base peak chromatograms of the crude ethanol extract (a), ethyl acetate fraction (b), and *n*-hexane fraction (c).

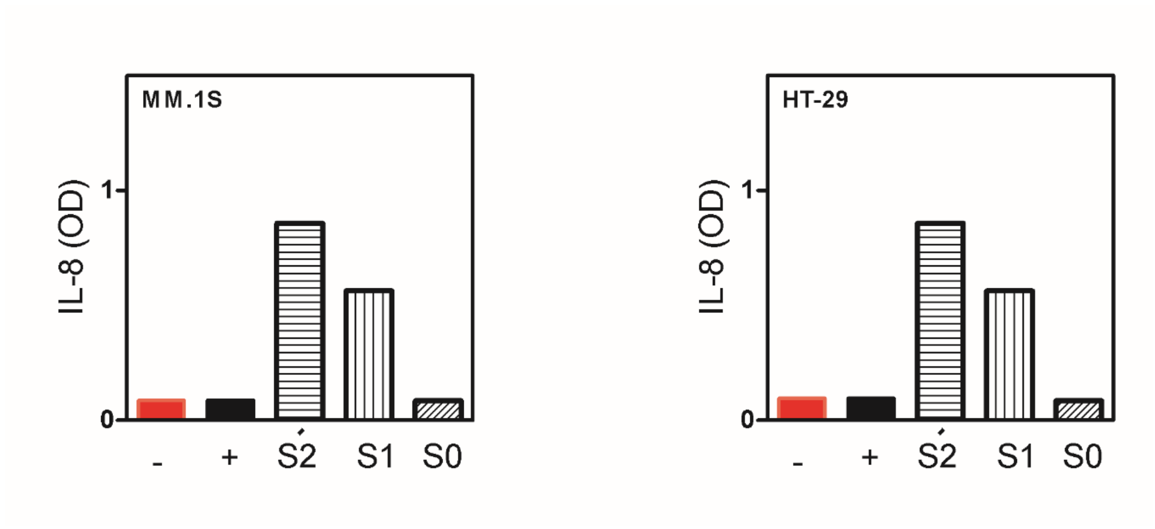

Fig.S2. Compound number **1** was not able to induce IL8 production in MM.1S and HT-29 cell lines. Cells were stimulated in triplicates with compound number 1. Next day, IL8 production was evaluated in the supernatant by ELISA. For control, IL8 standards with different concentrations 0, 1, and 2 ng/ml were used.

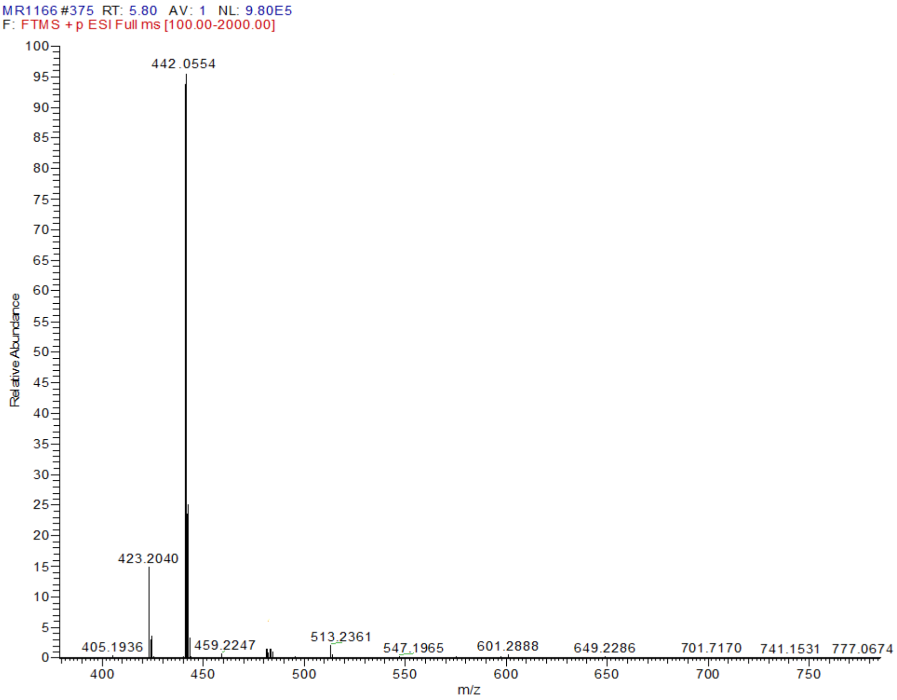

Fig.S3. HRESIMS spectrum of **1**.

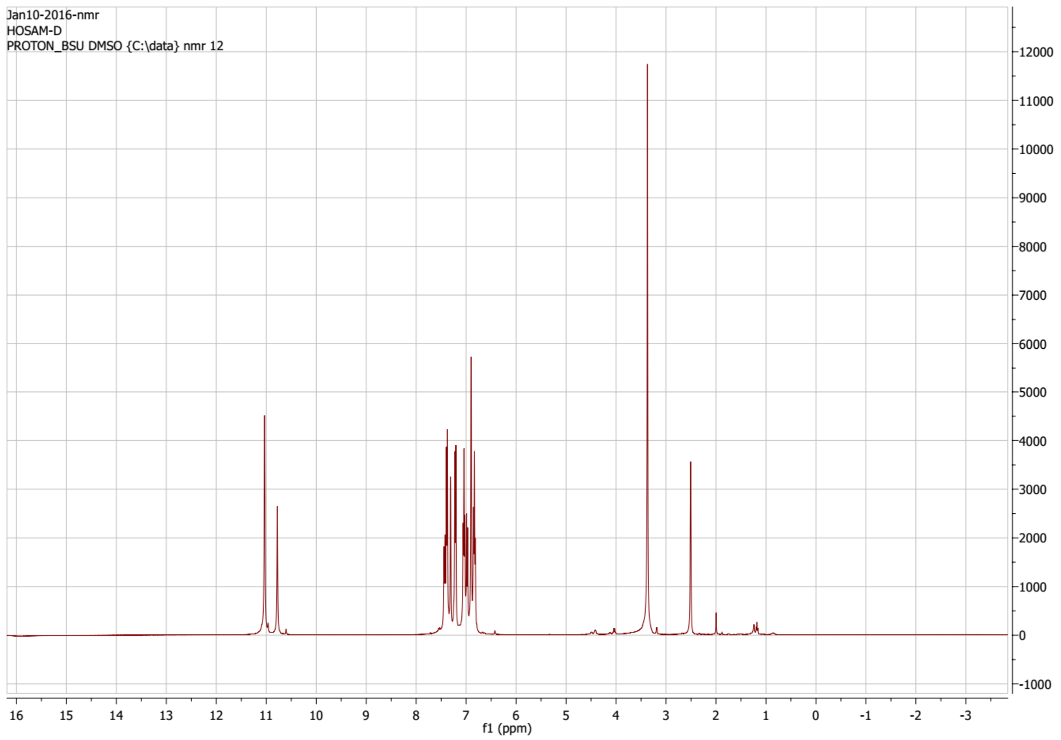

Fig.S4.^{1}H NMR spectrum (400 MHz, DMSO-d_6) of compound **1**.

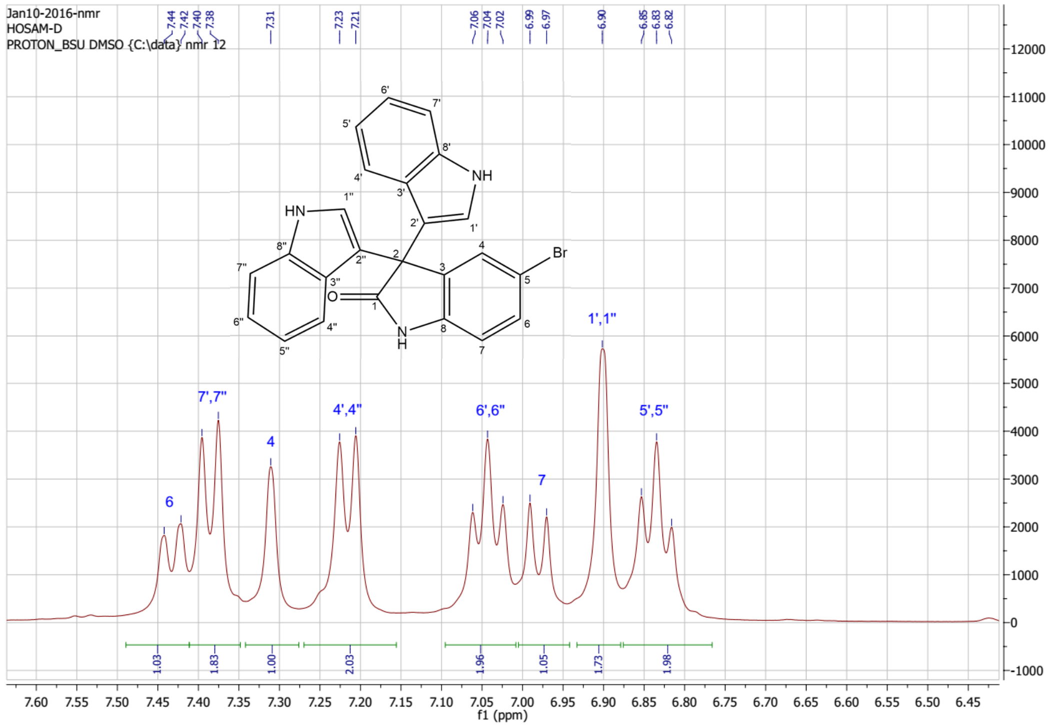

Fig.S5.^{1}H NMR (400 MHz, DMSO-d_6)spectrum expansion of compound **1.**

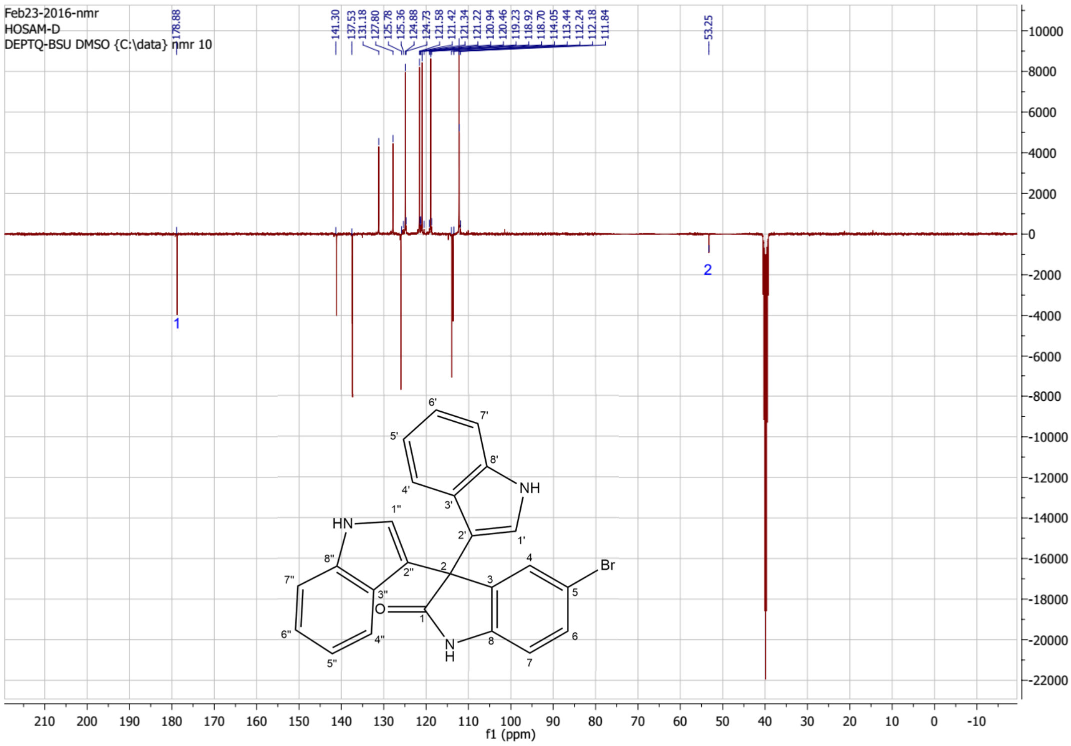

Fig.S6. DEPTQ (100 MHz, DMSO-d_6) spectrum of compound **1**.

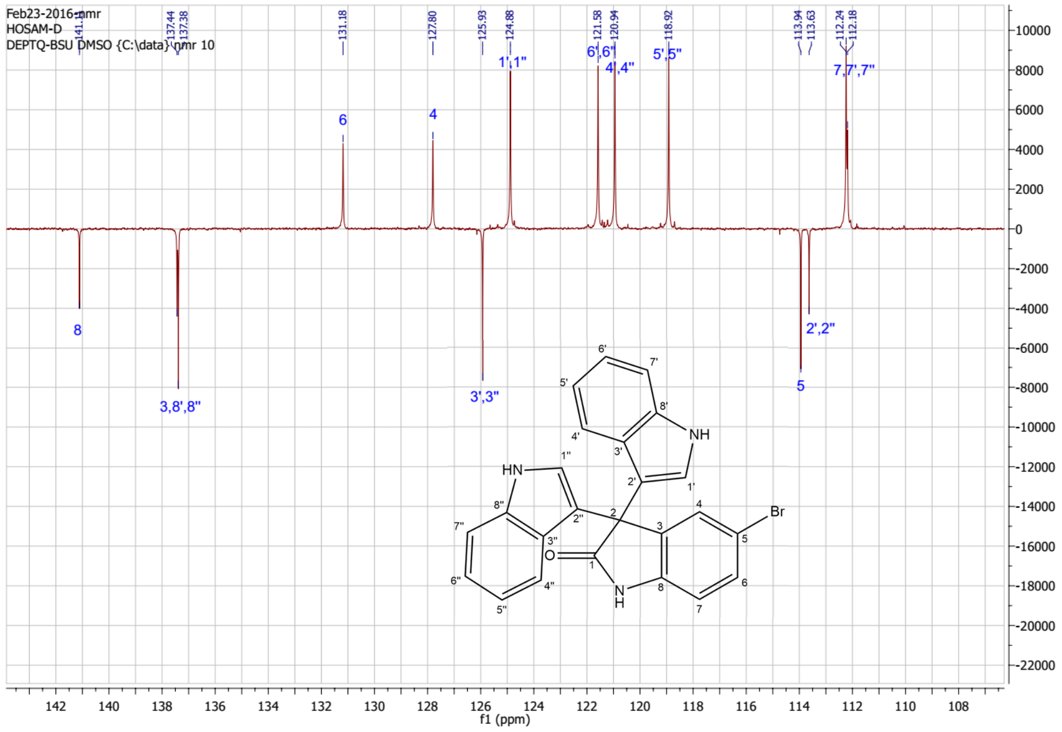

Fig.S7. DEPTQ (100 MHz, DMSO-d_6) spectrum expansion of compound **1**.

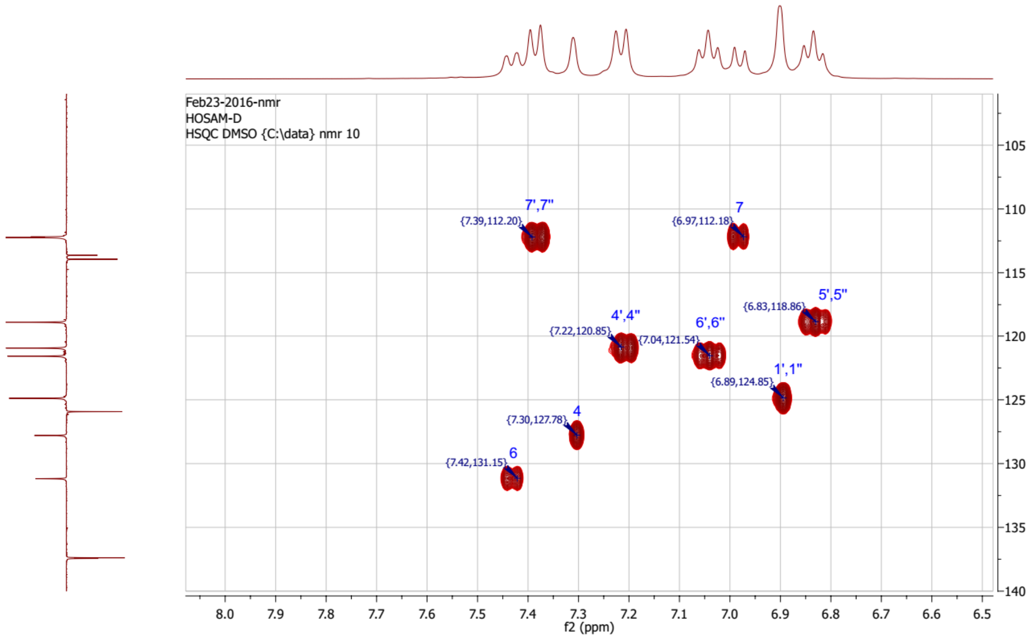

Fig.S8. HSQC spectrum expansion of compound **1**.

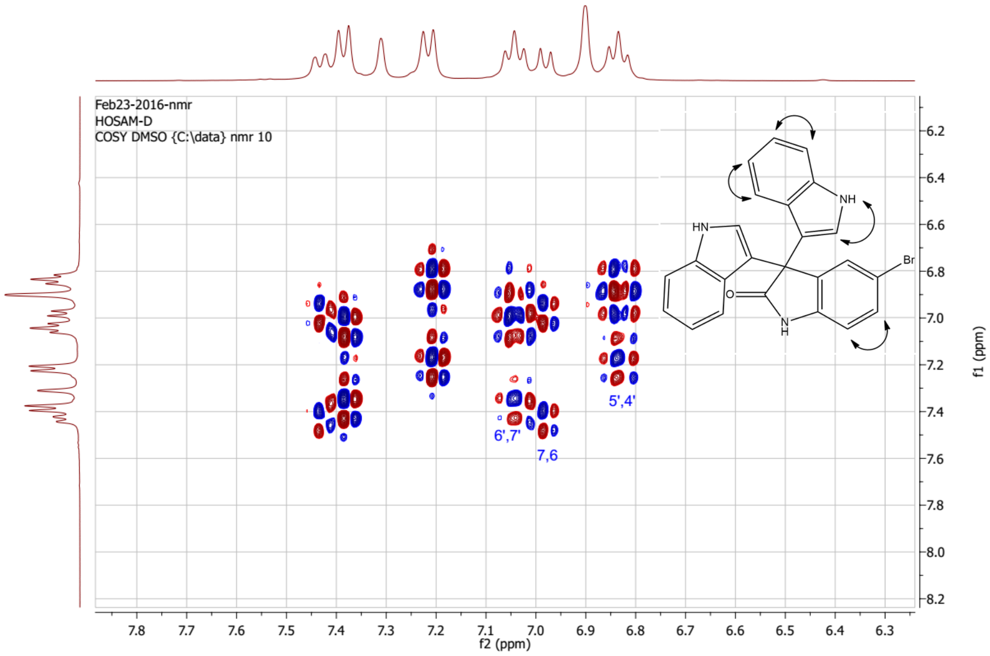

Fig.S9. ^{1}H-^{1}H COSY spectrum expansion of compound **1**.

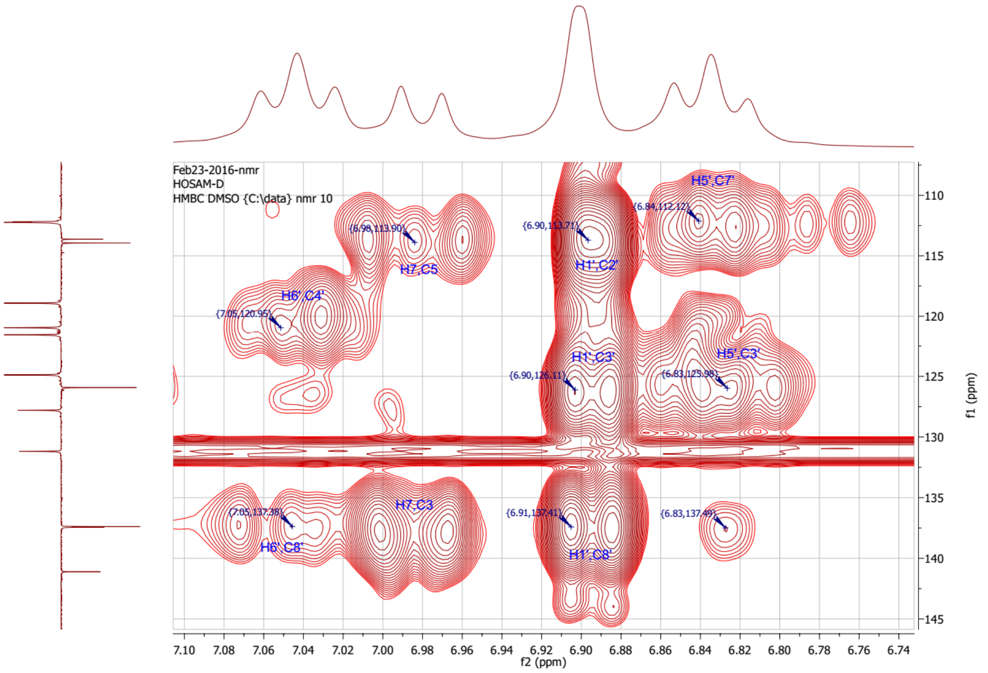

Fig.S10. HMBC spectrum of compound **1**.

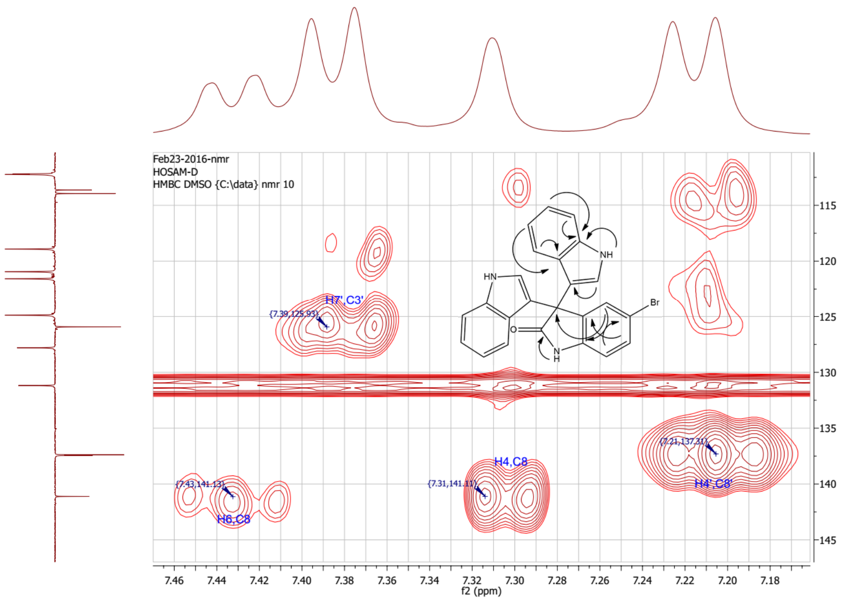

Fig.S11. HMBC spectrum expansion of compound **1**.

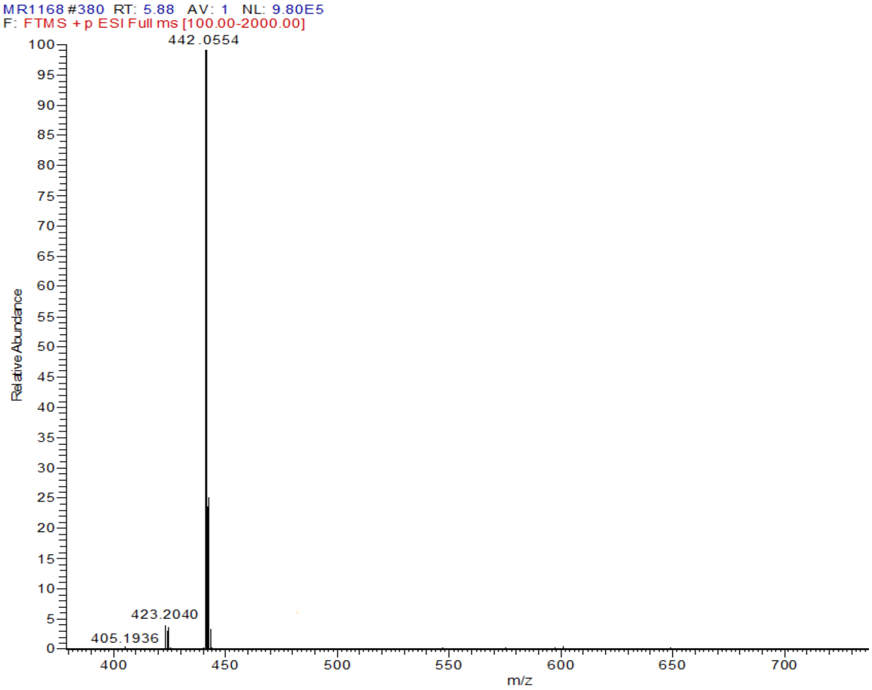

Fig.12S. HRESIMS spectrum of **2**.

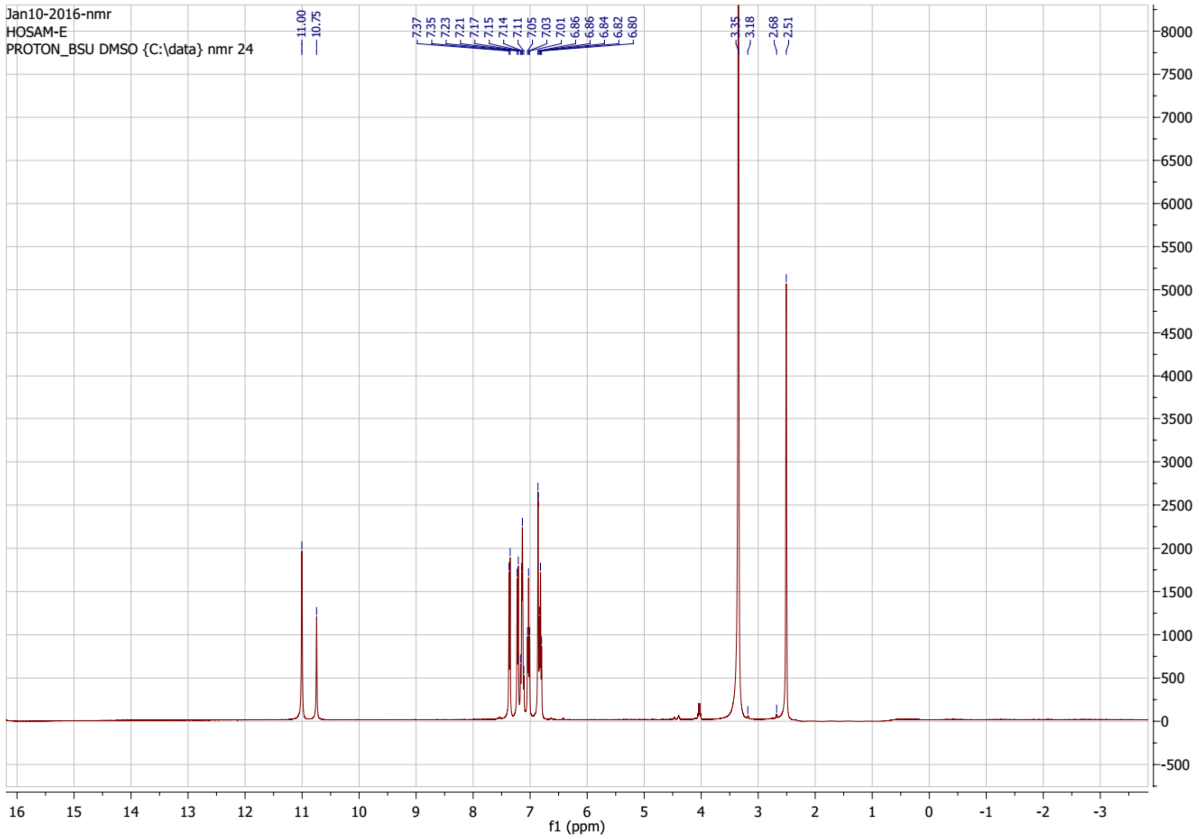

Fig.S13.^{1}H NMR (400 MHz, DMSO-d_6) spectrum of compound **2**.

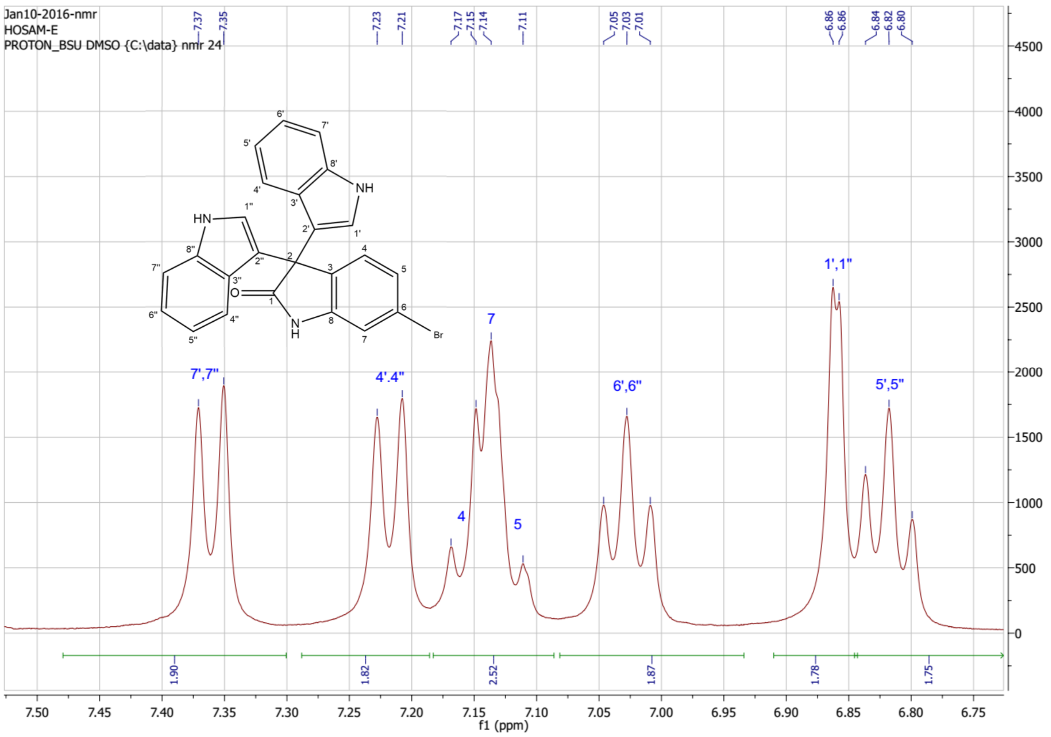

Fig.S14. ^{1}H NMR (400 MHz, DMSO-d_6) spectrum expansion of compound **2**.

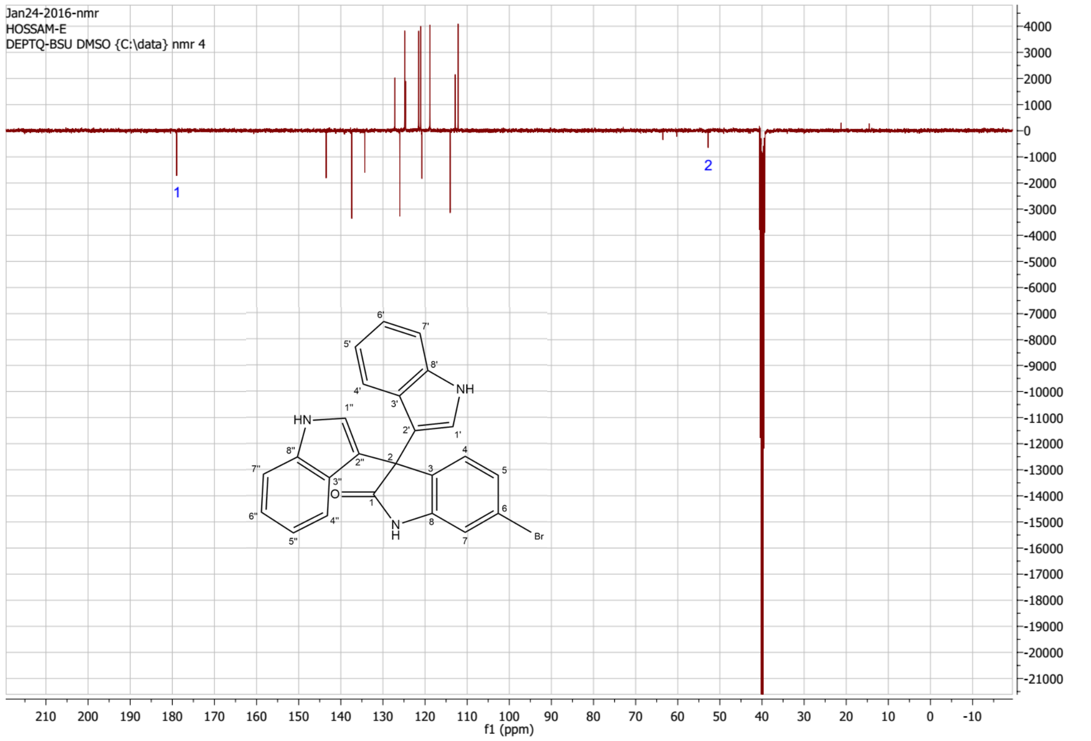

Fig.S15. DEPTQ (100 MHz, DMSO-d_6) spectrum of compound **2**.

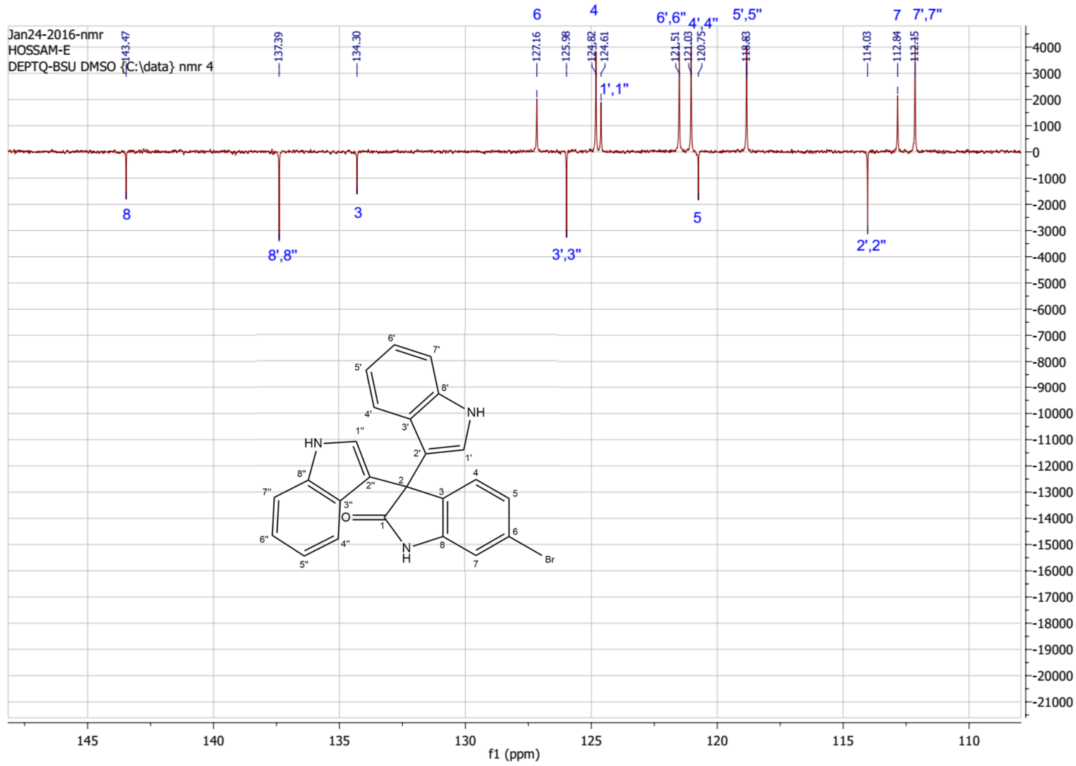

Fig.S16. DEPTQ (100 MHz, DMSO-d_6) spectrum expansion of compound **2.**

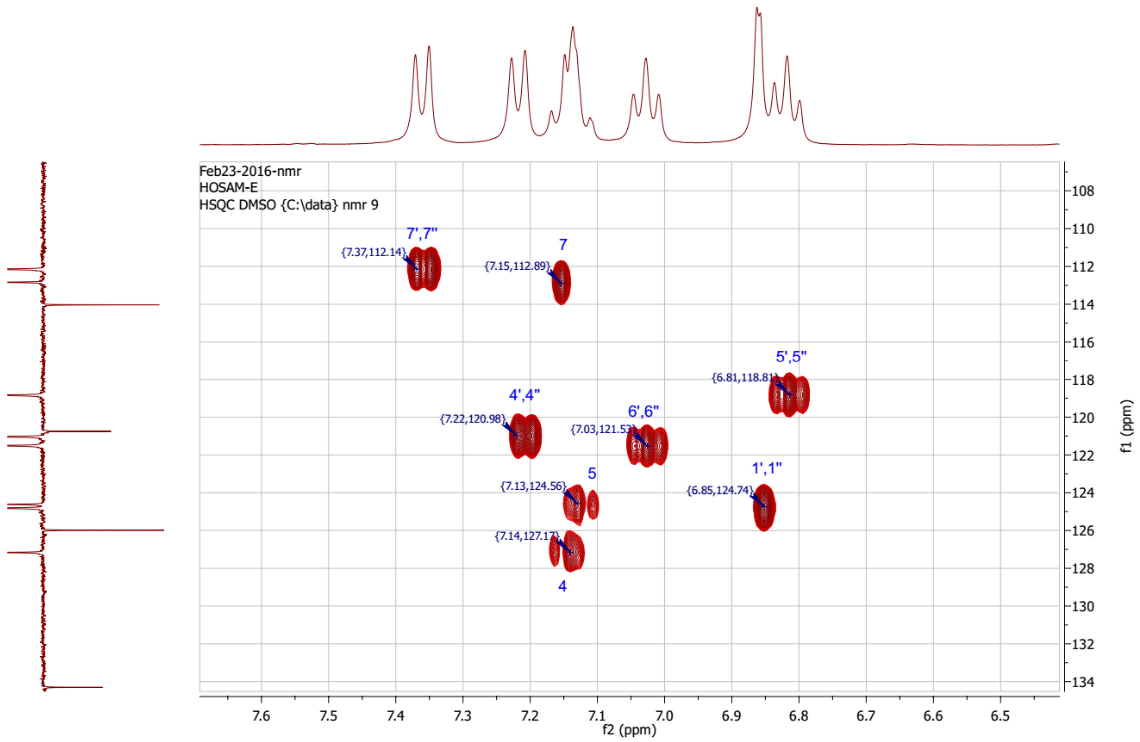

Fig.S17. HSQC spectrum expansion of compound **2**.

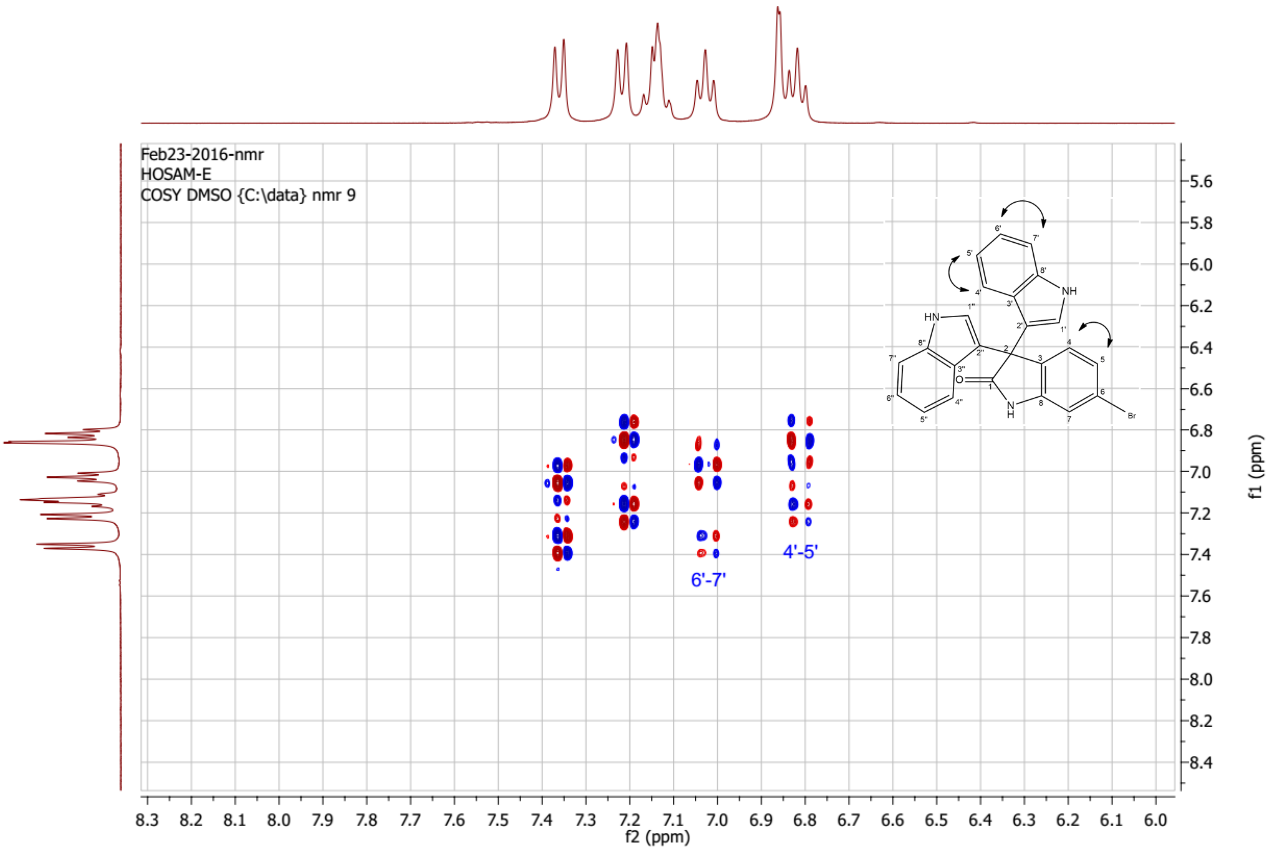

Fig.S18. ^{1}H-^{1}H COSY spectrum expansion of compound **2**.

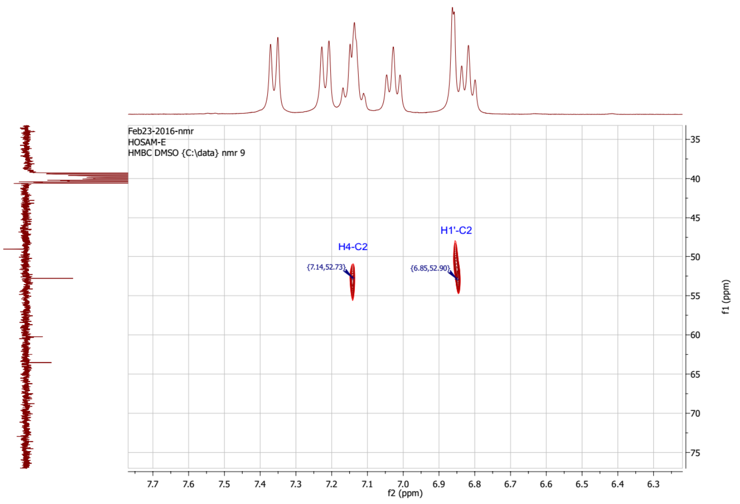

Fig.S19. HMBC spectrum 1st expansion of compound **2**.

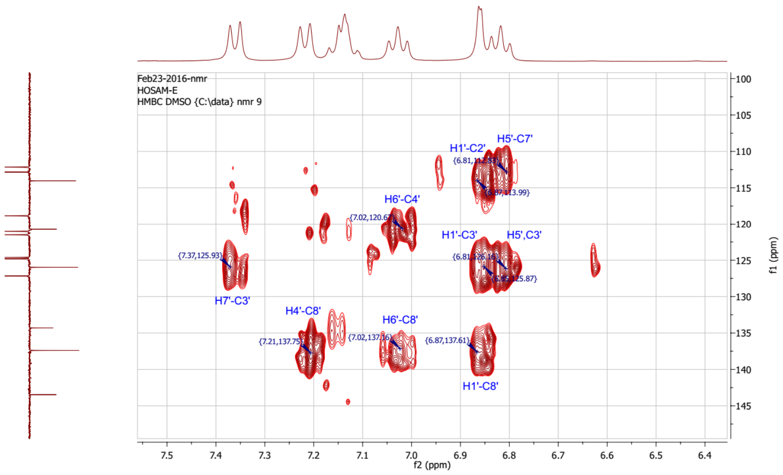

Fig.S20. HMBC spectrum 2nd expansion of compound **2**.

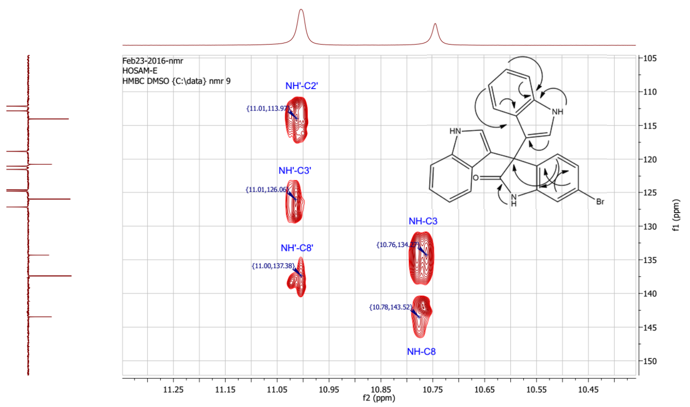

Fig.S21. HMBC spectrum 3rd expansion of compound **2**.

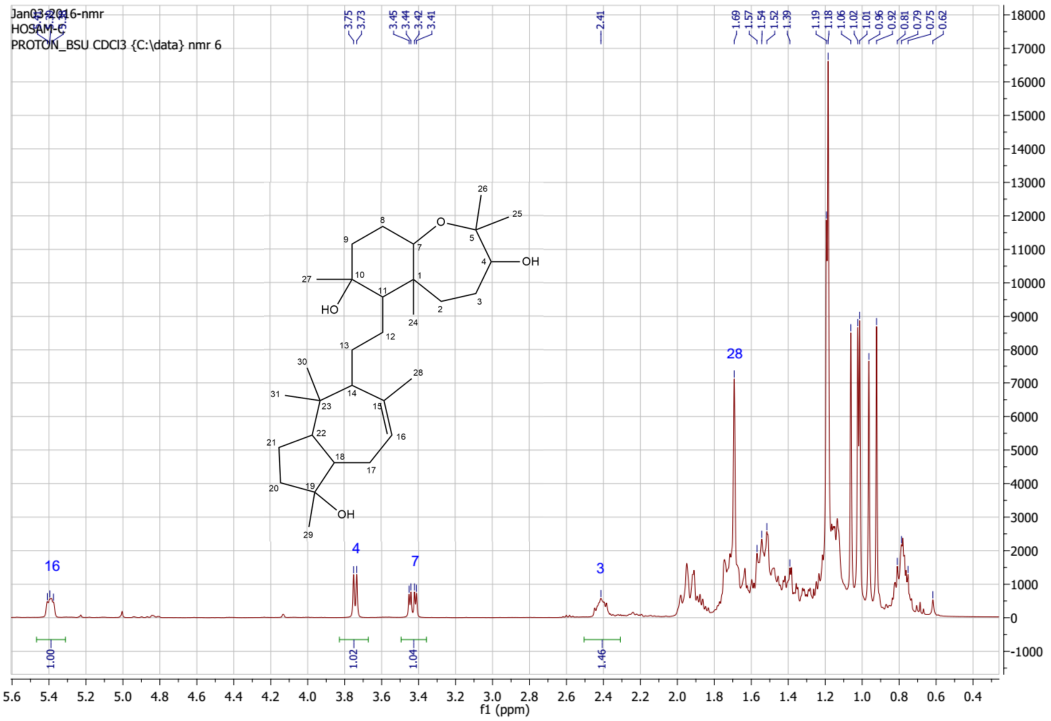

Fig.S22.^{1}H NMR (400 MHz, $CDCl_3$) spectrum of compound **3**.

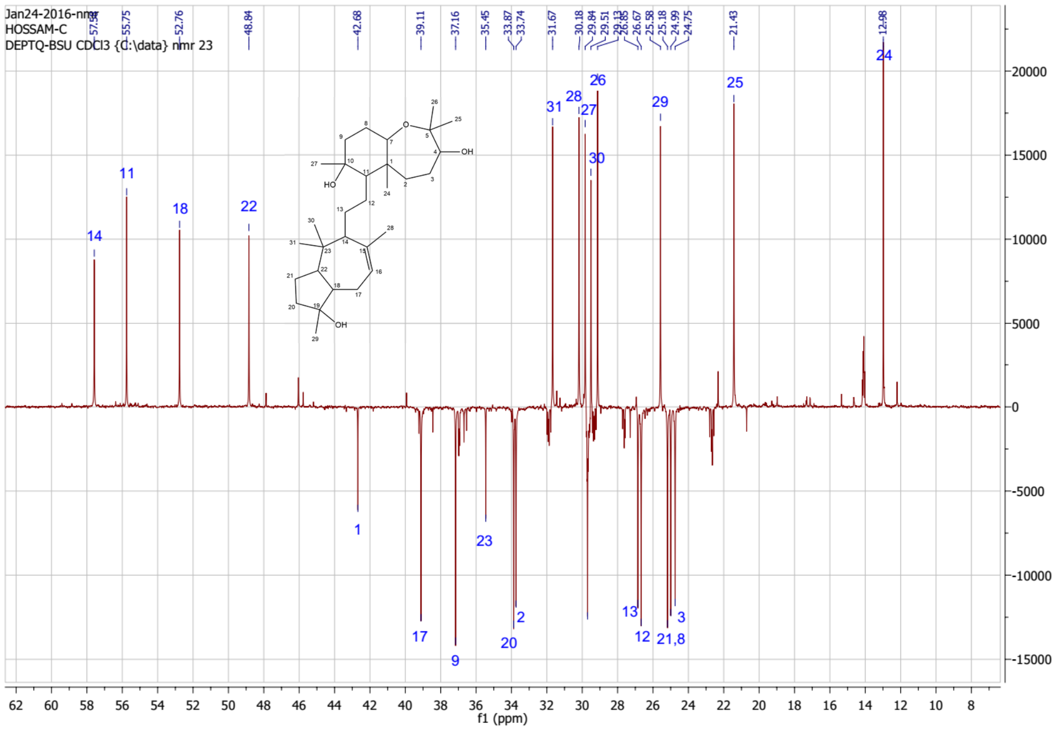

Fig.S23. DEPTQ (100 MHz, CDCl$_3$) spectrum of compound **3**.

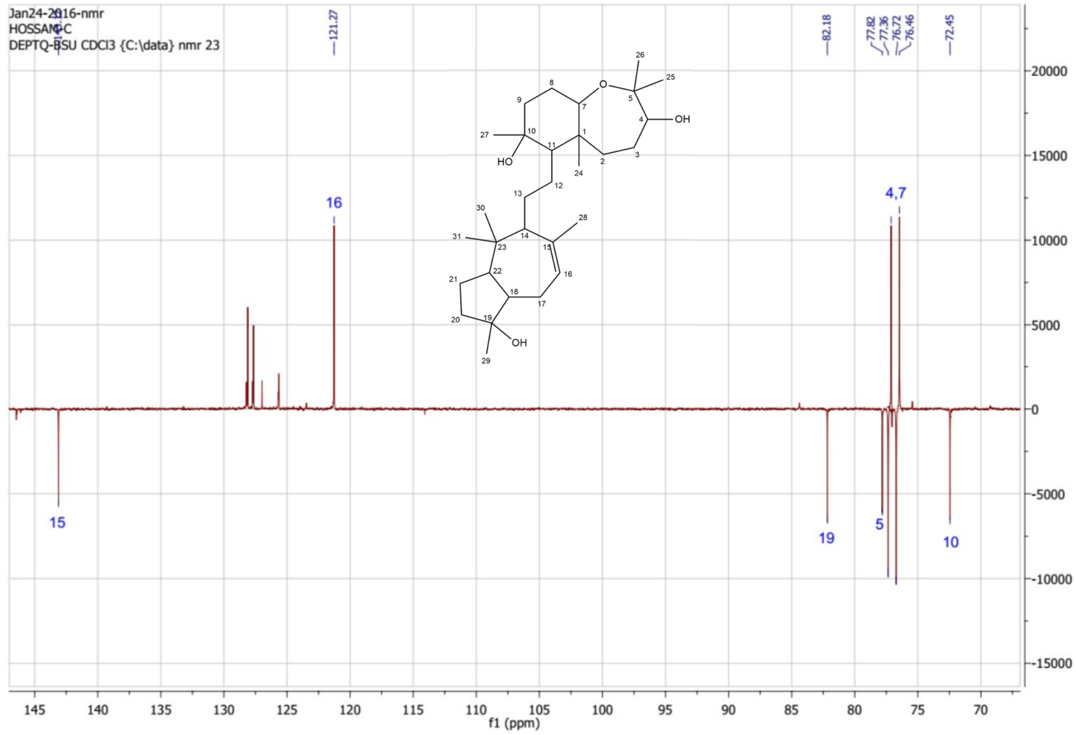

Fig.S24. DEPTQ (100 MHz, $CDCl_3$) spectrum expansion of compound **3**.

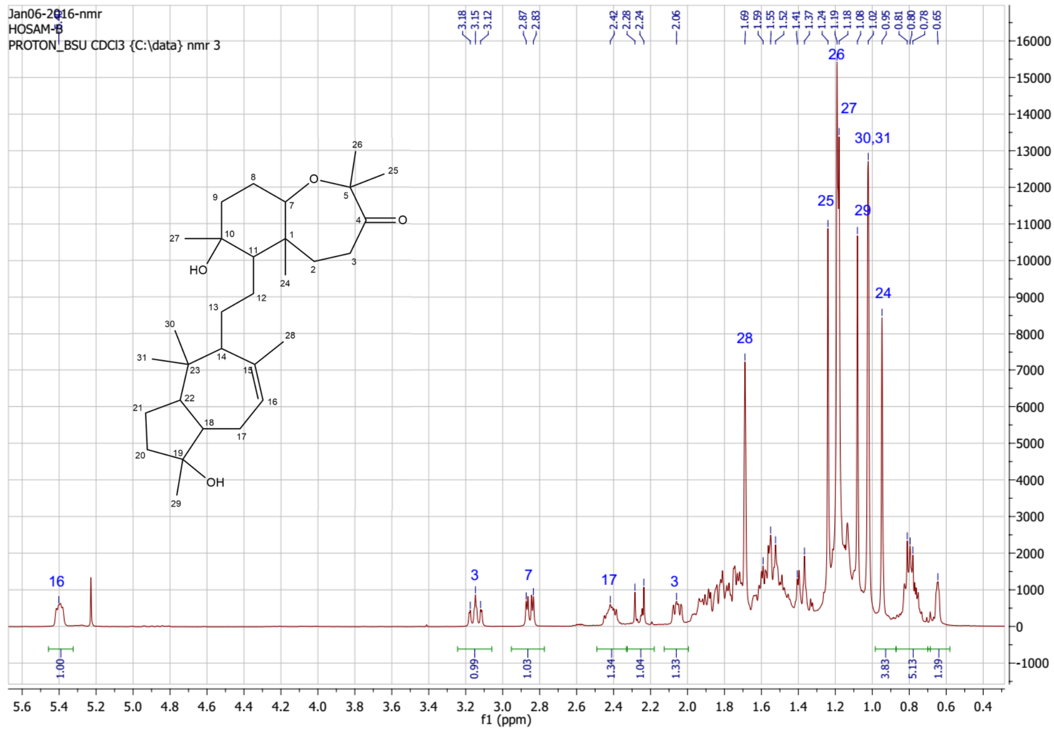

Fig.S25.^{1}H NMR (400 MHz, $CDCl_3$) spectrum of compound **4**.

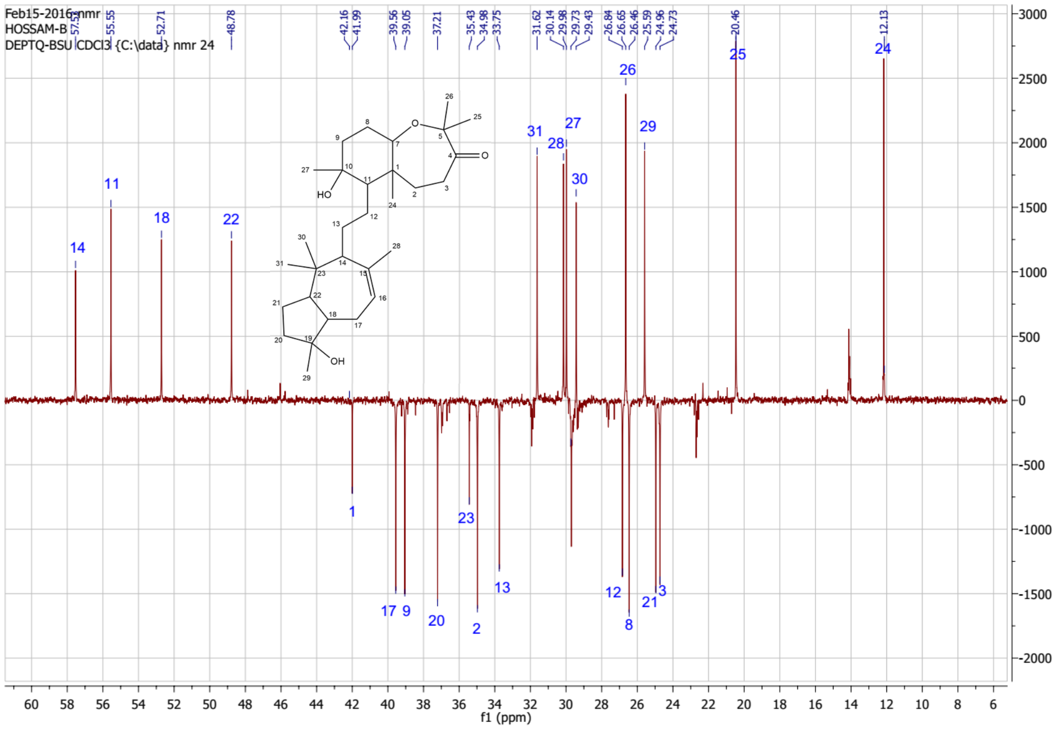

Fig.S26. DEPTQ (100 MHz, $CDCl_3$) spectrum of compound **4**.

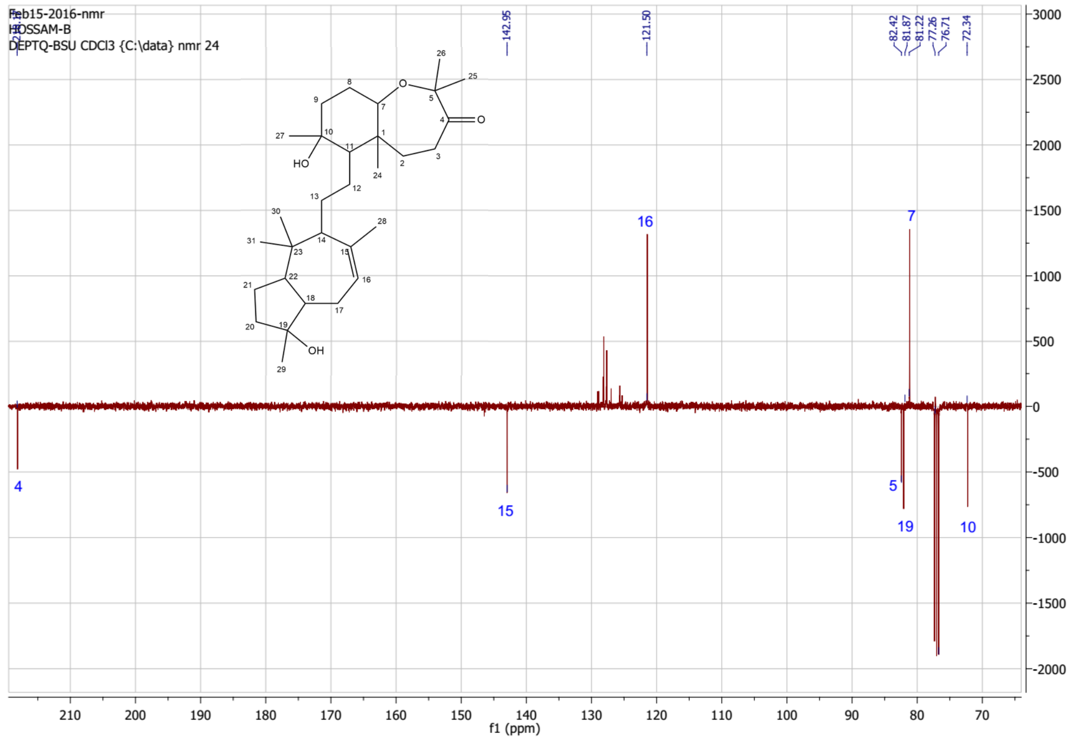

Fig.S27. DEPTQ (100 MHz, $CDCl_3$) spectrum expansion of compound **4**.

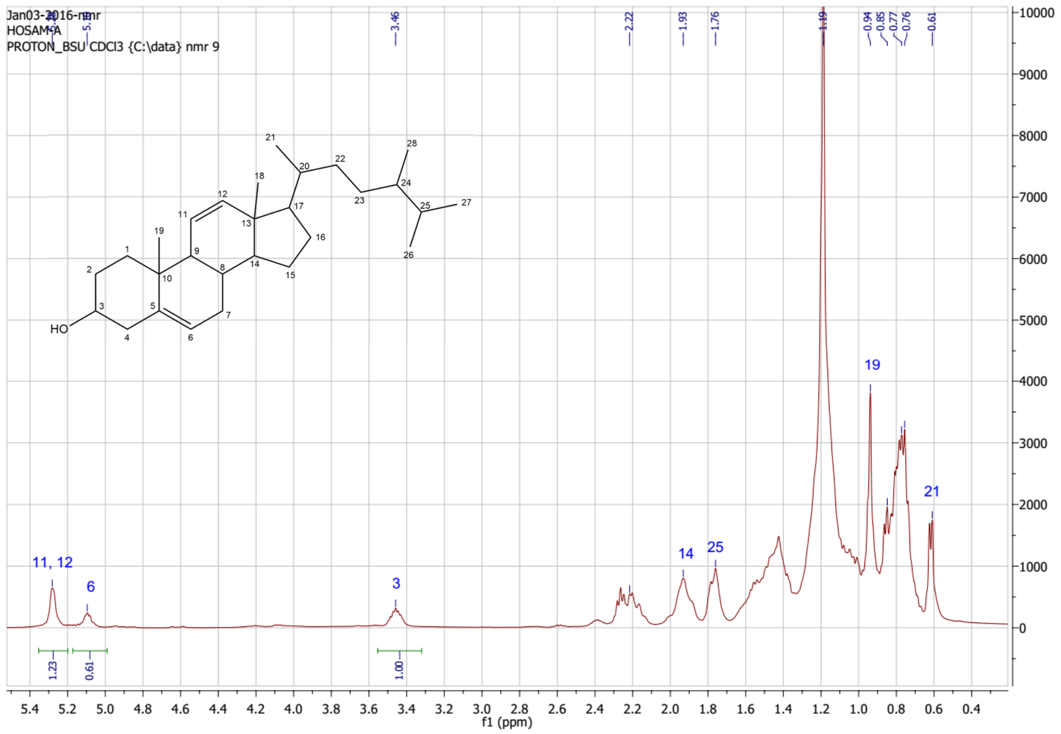

Fig.S28. ^{1}H NMR (400 MHz, $CDCl_3$) spectrum of compound **5**.

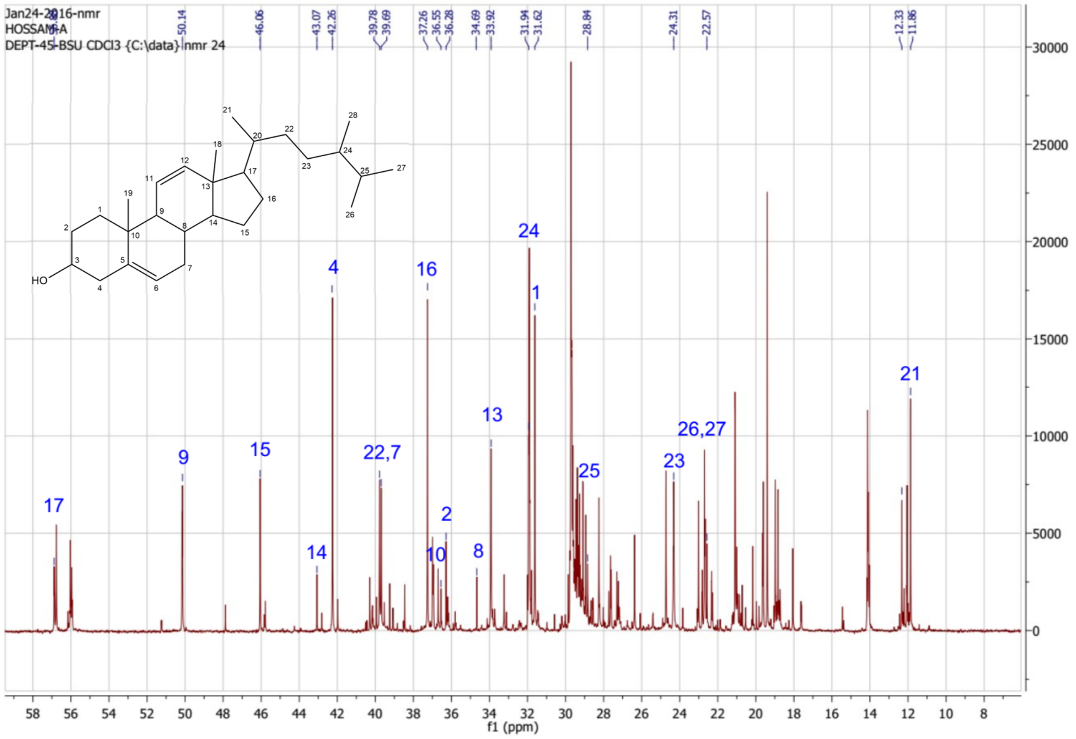

Fig.S29. ^{13}C NMR (100 MHz, $CDCl_3$) spectrum of compound **5.**

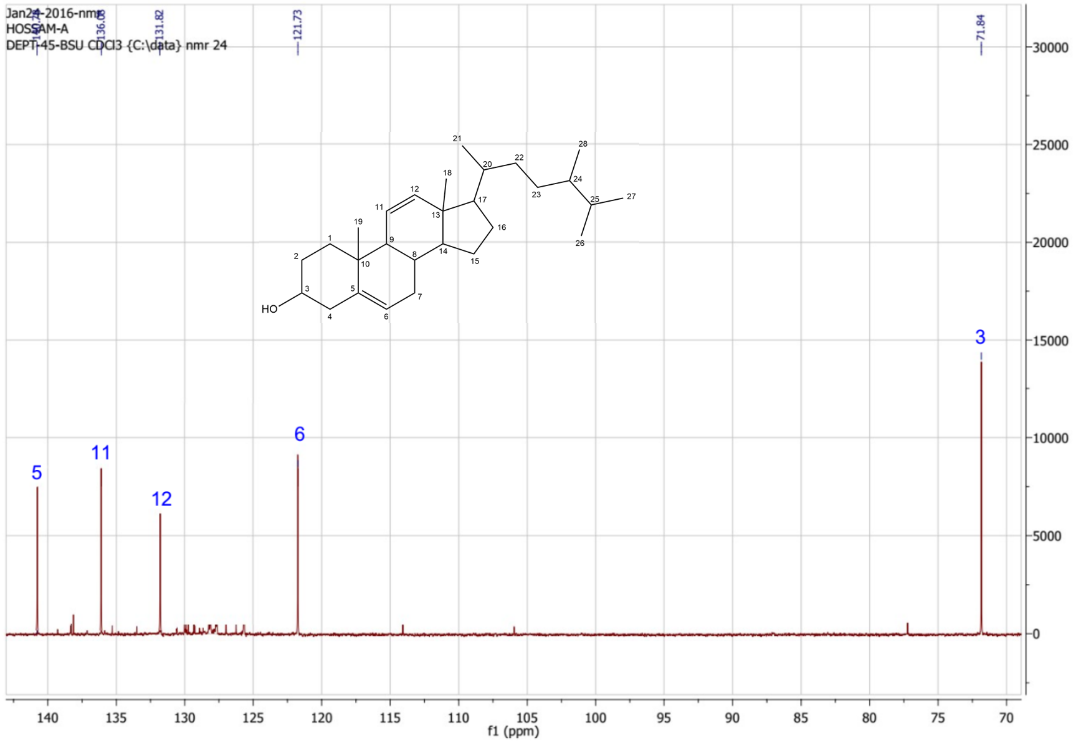

Fig.S30. ^{13}C NMR (100 MHz, $CDCl_3$) spectrum expansion of compound **5.**

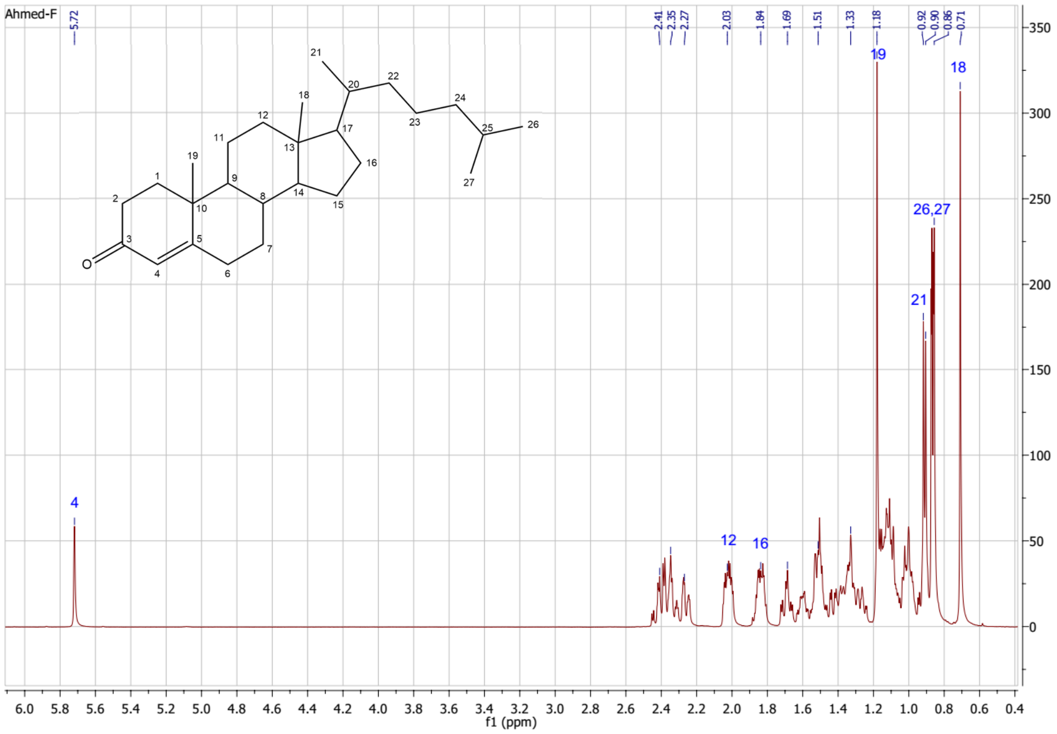

Fig.S31. ^{1}H NMR (400 MHz, $CDCl_3$) spectrum of compound **6.**

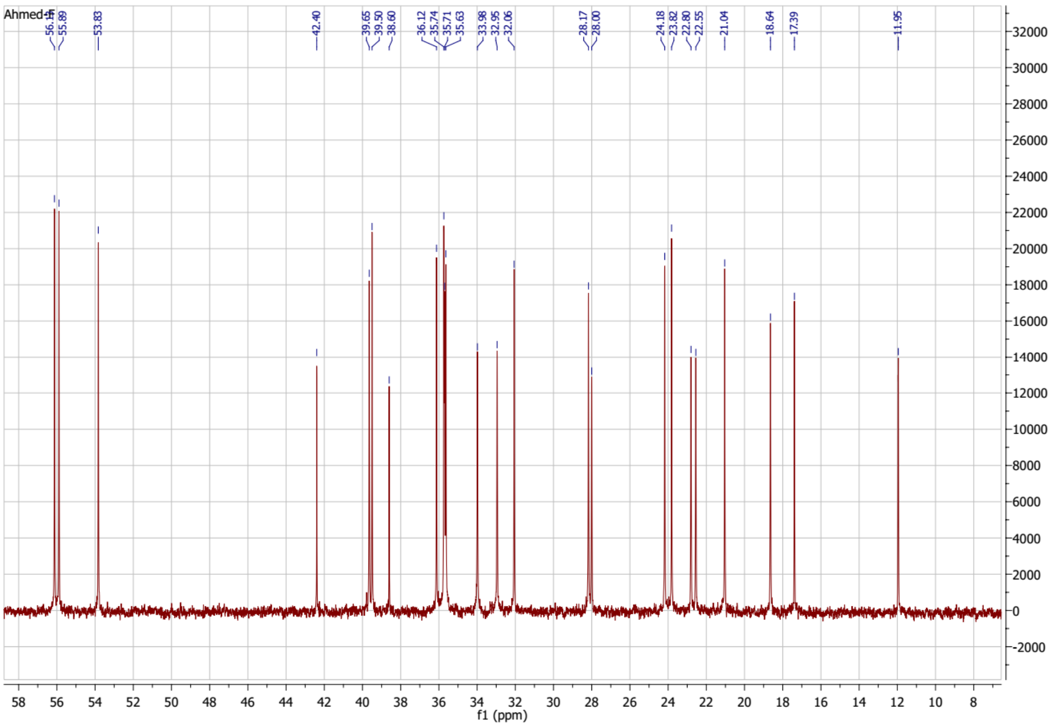

Fig.S32. ^{13}C NMR (100 MHz, $CDCl_3$) spectrum expansion of compound **6.**

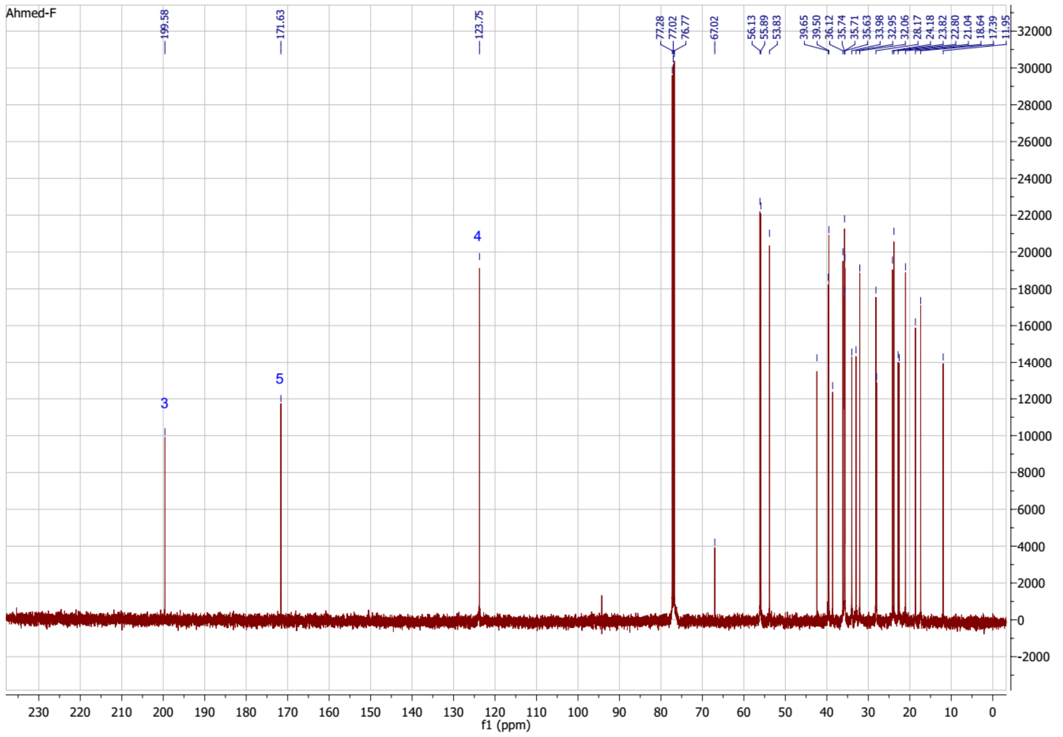

Fig.S33. ^{13}C NMR (400 MHz, $CDCl_3$) spectrum expansion of compound **6.**

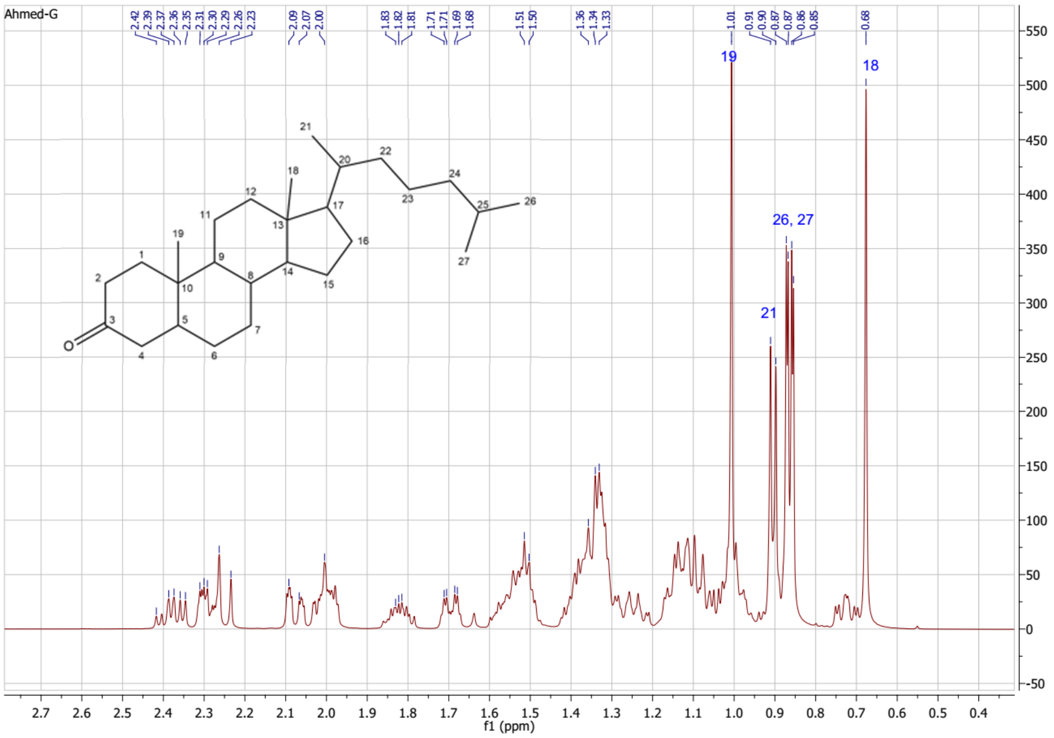

Fig.S34. ^{1}H NMR (400 MHz, $CDCl_3$) spectrum of compound **7.**

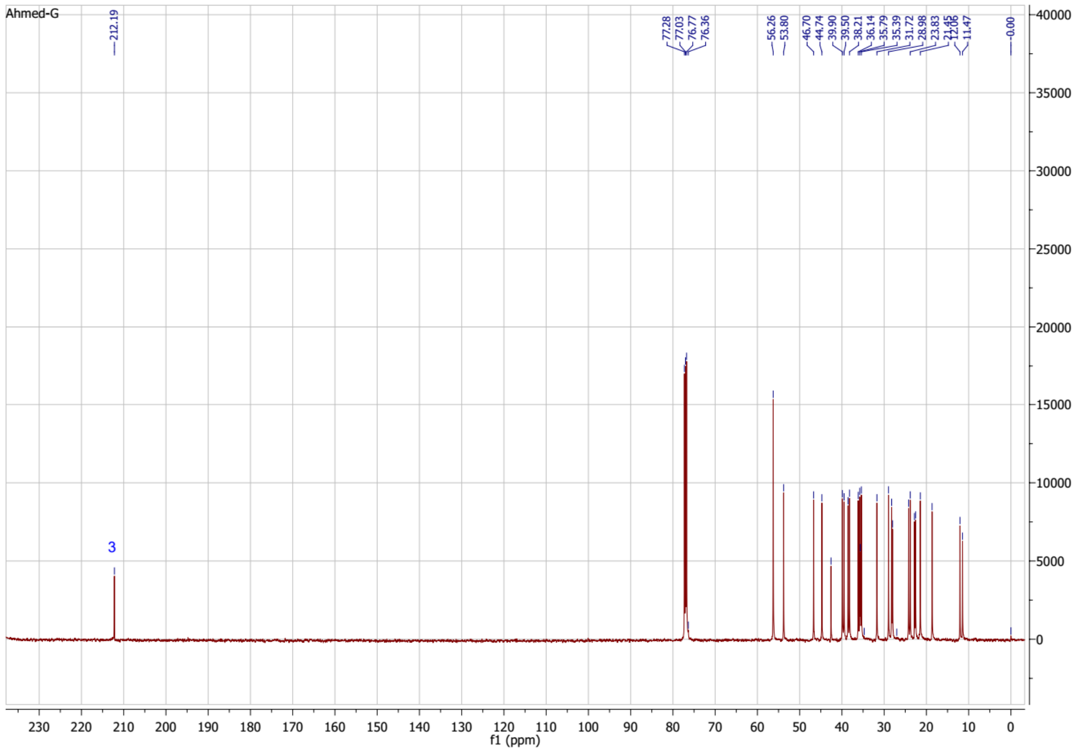

Fig.S35. ^{13}C NMR (100 MHz, $CDCl_3$) spectrum expansion of compound **7.**

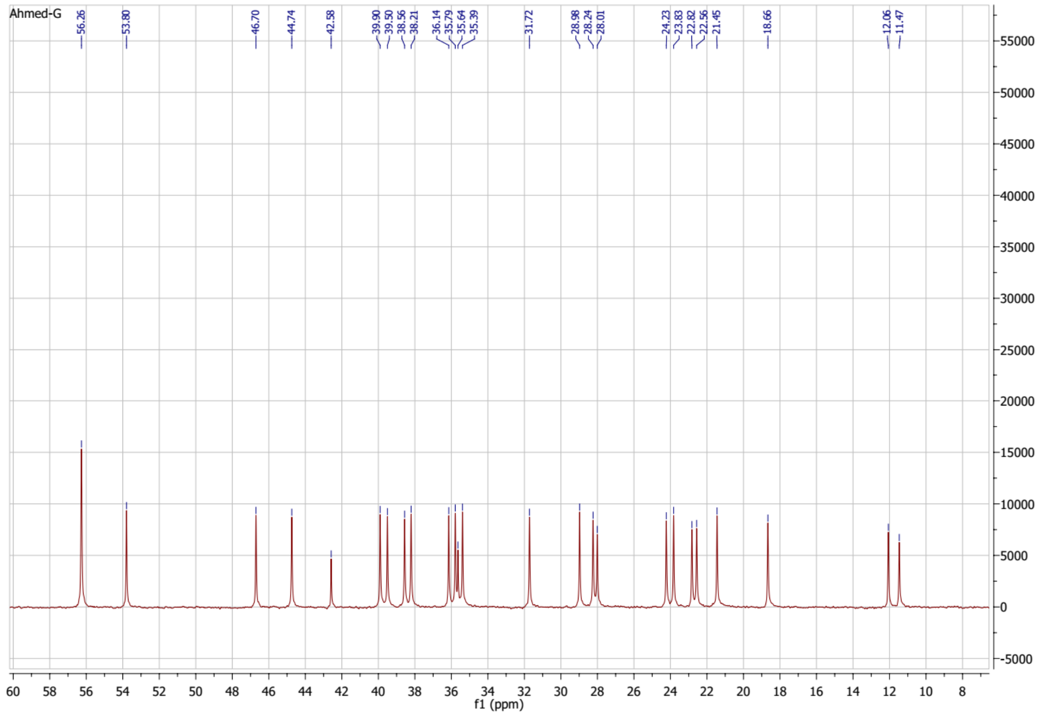

Fig.S36. ^{13}C NMR (100 MHz, $CDCl_3$) spectrum expansion of compound **7.**

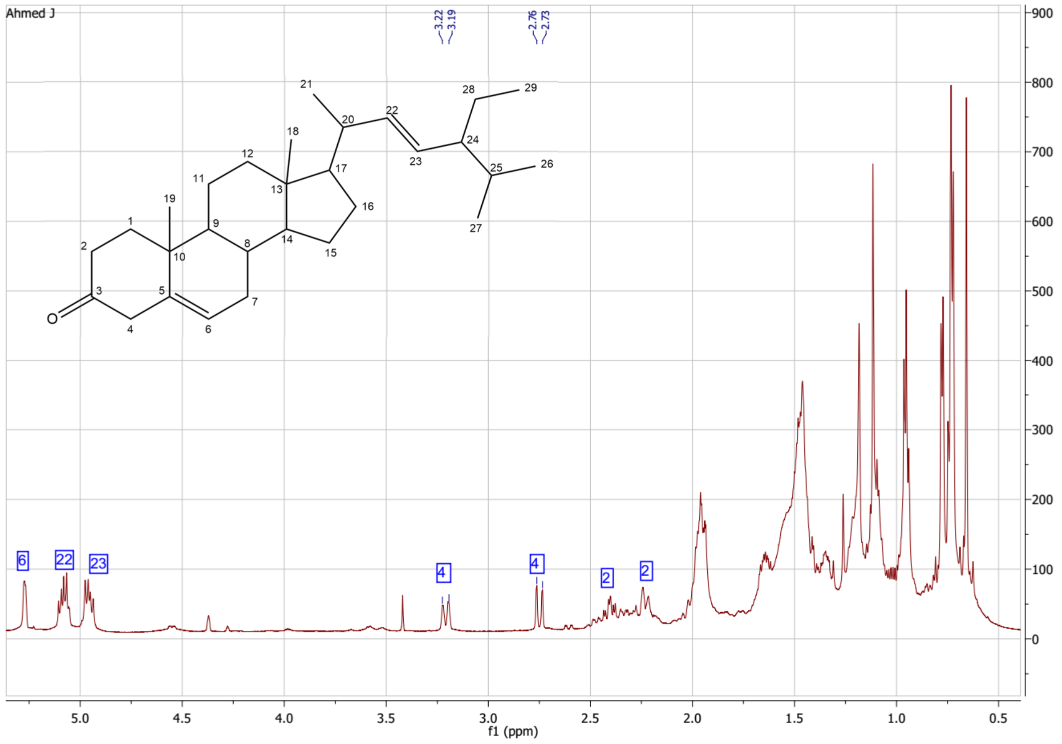

Fig.S37. ^{1}H NMR (400 MHz, $CDCl_3$) spectrum of compound **8.**

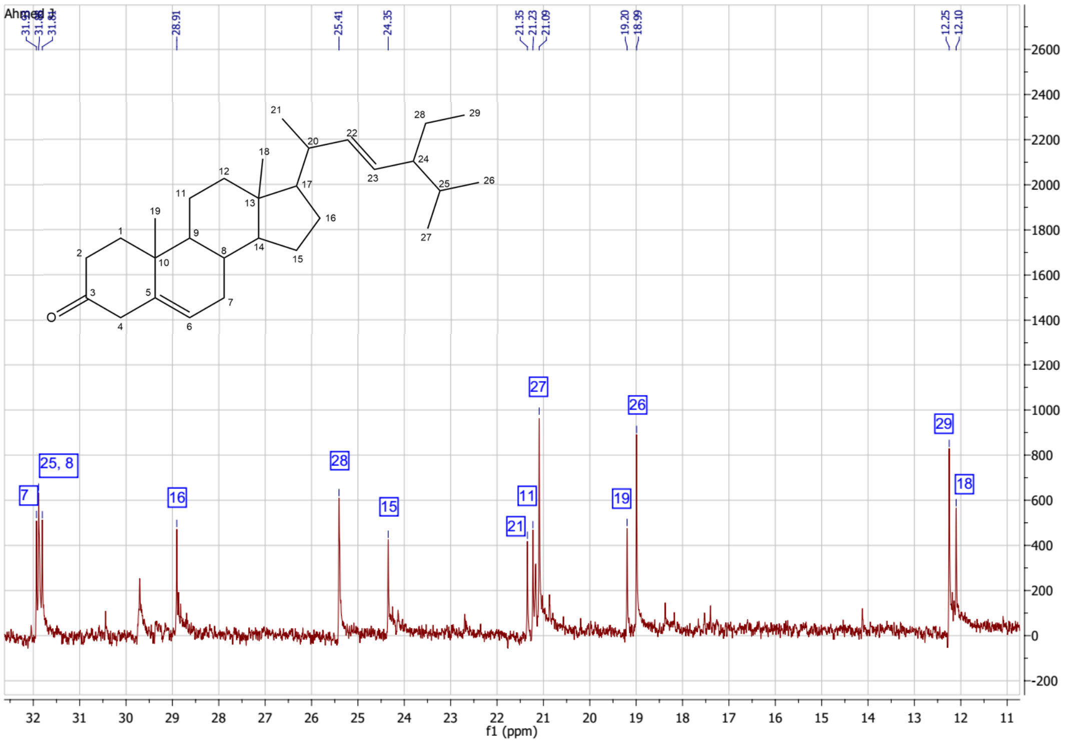

Fig.S38. ^{13}C NMR (100 MHz, $CDCl_3$) spectrum expansion of compound **8.**

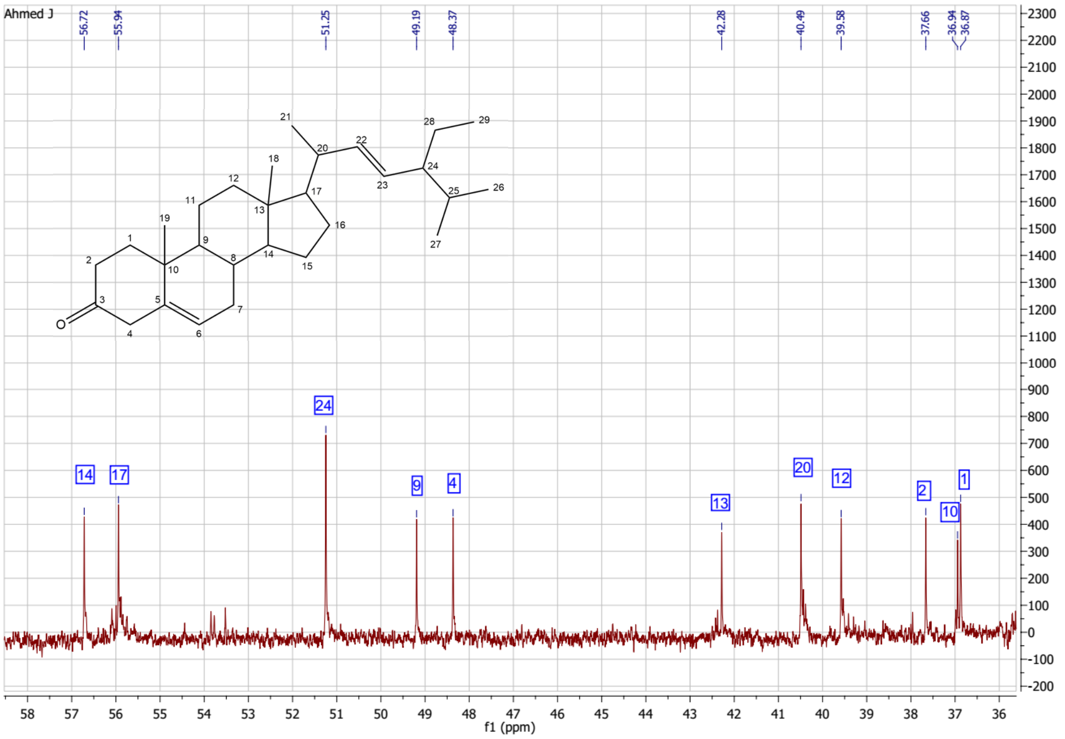

Fig.S39. ^{13}C NMR (100 MHz, $CDCl_3$) spectrum expansion of compound **8.**

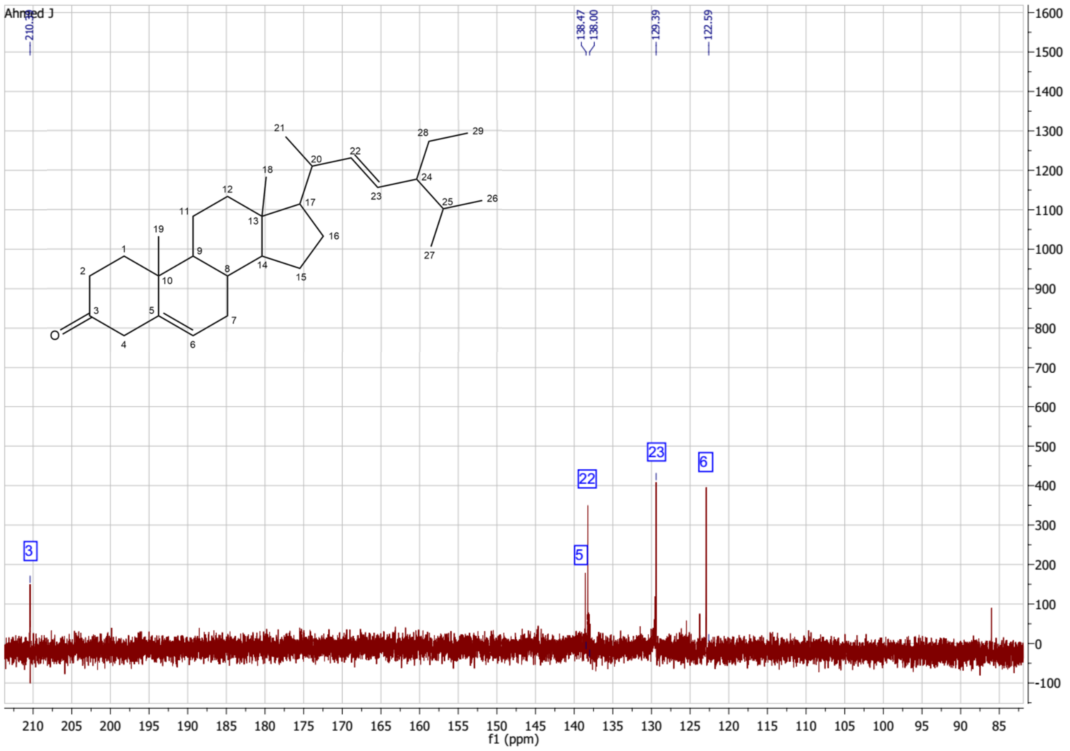

Fig.S40. ^{13}C NMR (100 MHz, $CDCl_3$) spectrum expansion of compound **8.**

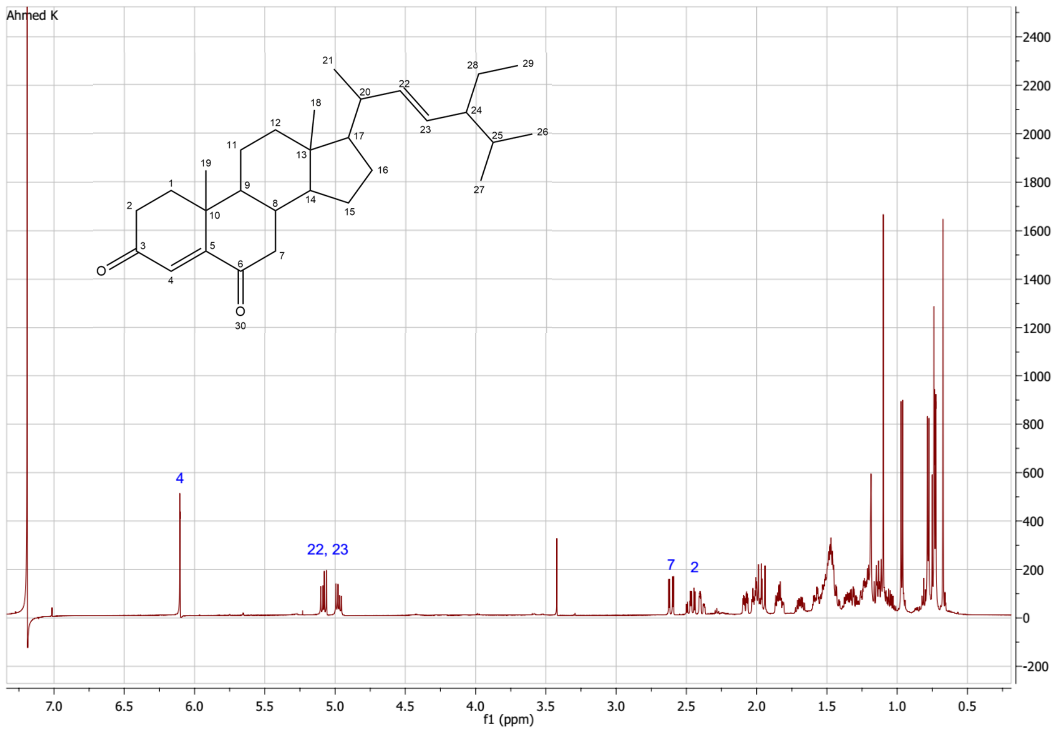

Fig.S41. ^{1}H NMR (400 MHz, $CDCl_3$) spectrum of compound **9.**

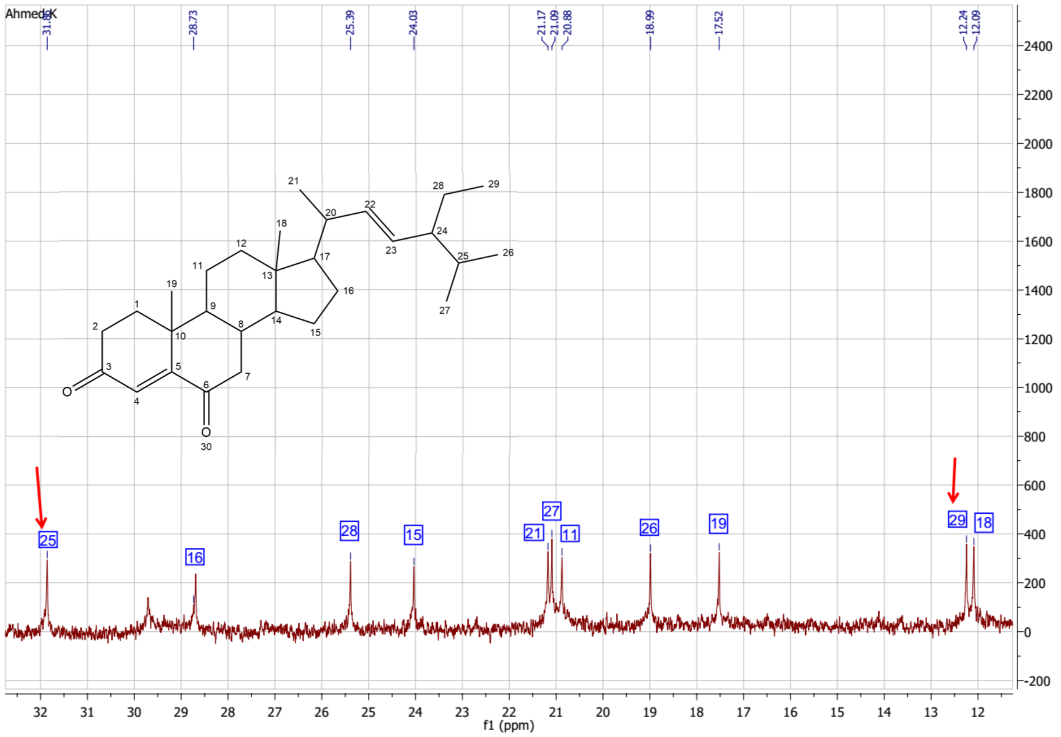

Fig.S42. ^{13}C NMR (100 MHz, CDCl$_3$) spectrum expansion of compound **9.**

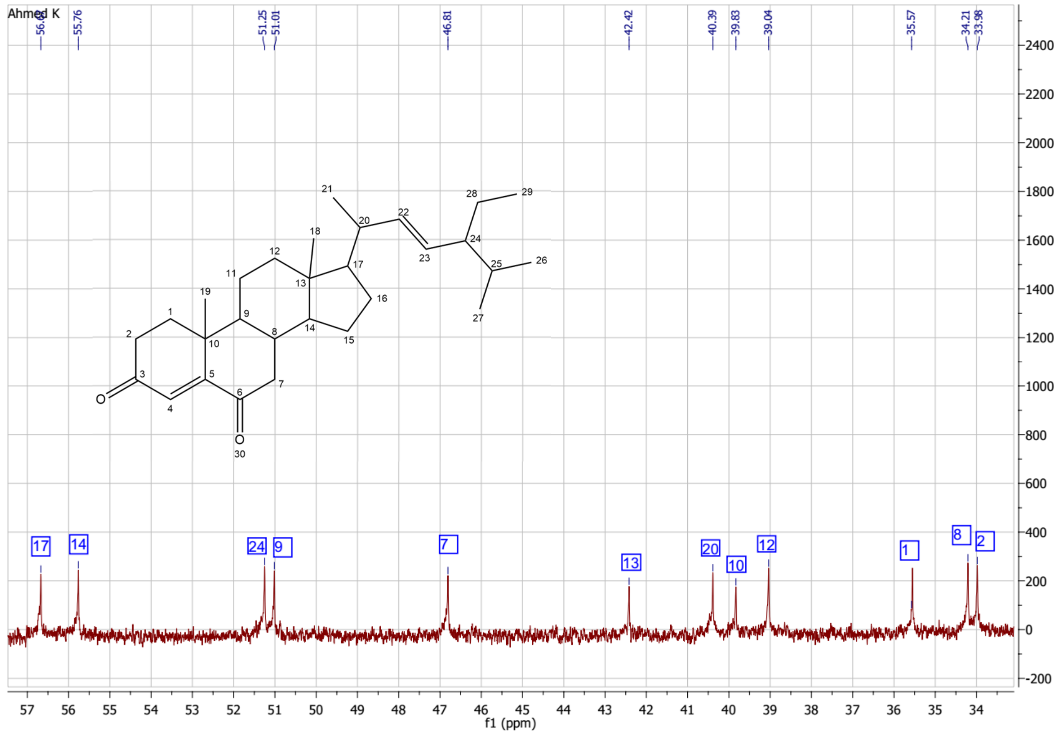

Fig.S43. ^{13}C NMR (100 MHz, $CDCl_3$) spectrum expansion of compound **9.**

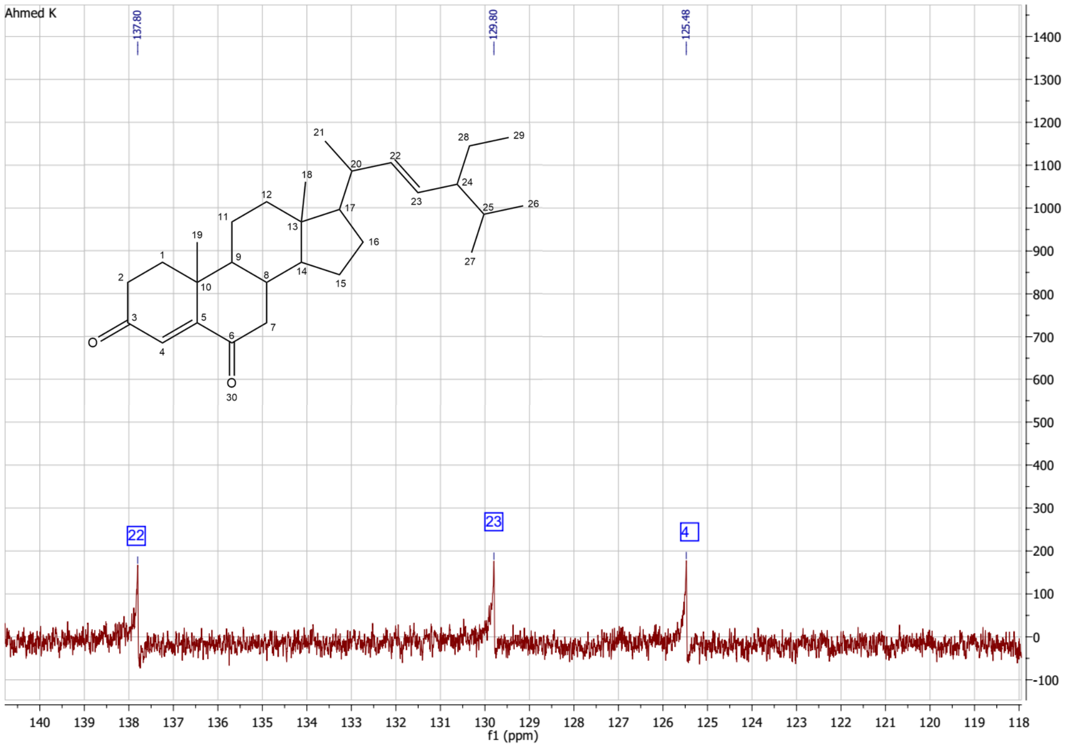

Fig.S44. ^{13}C NMR (100 MHz, $CDCl_3$) spectrum expansion of compound **9.**

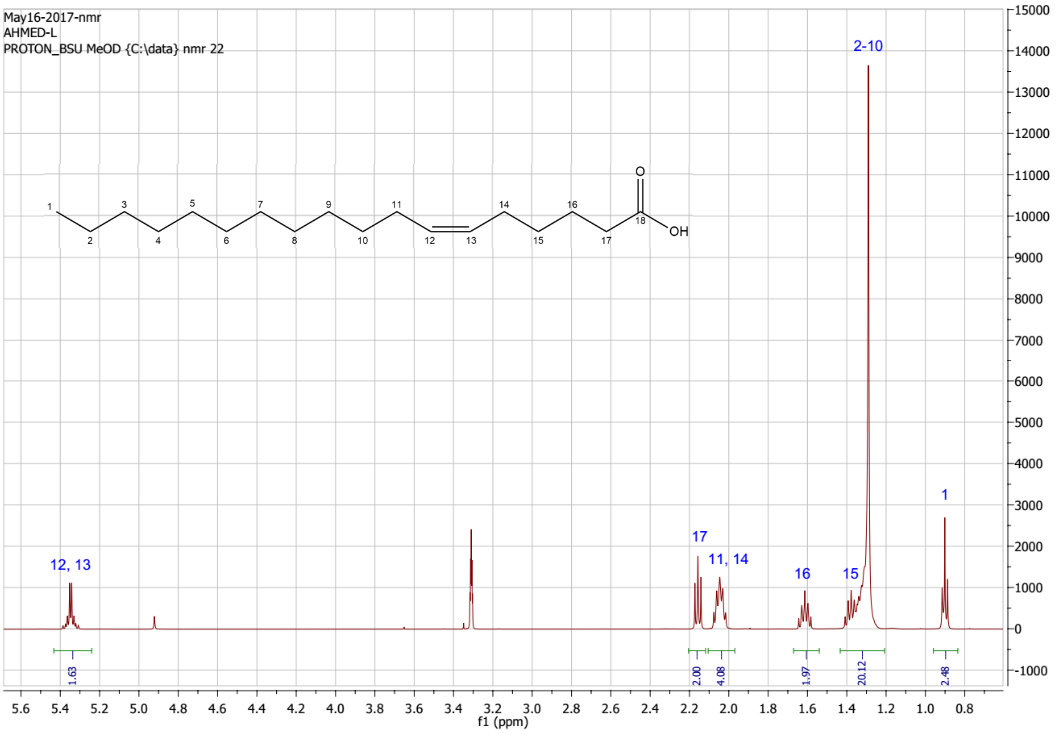

Fig.S45. ^{1}H NMR (400 MHz, $CDCl_3$) spectrum of compound **10.**

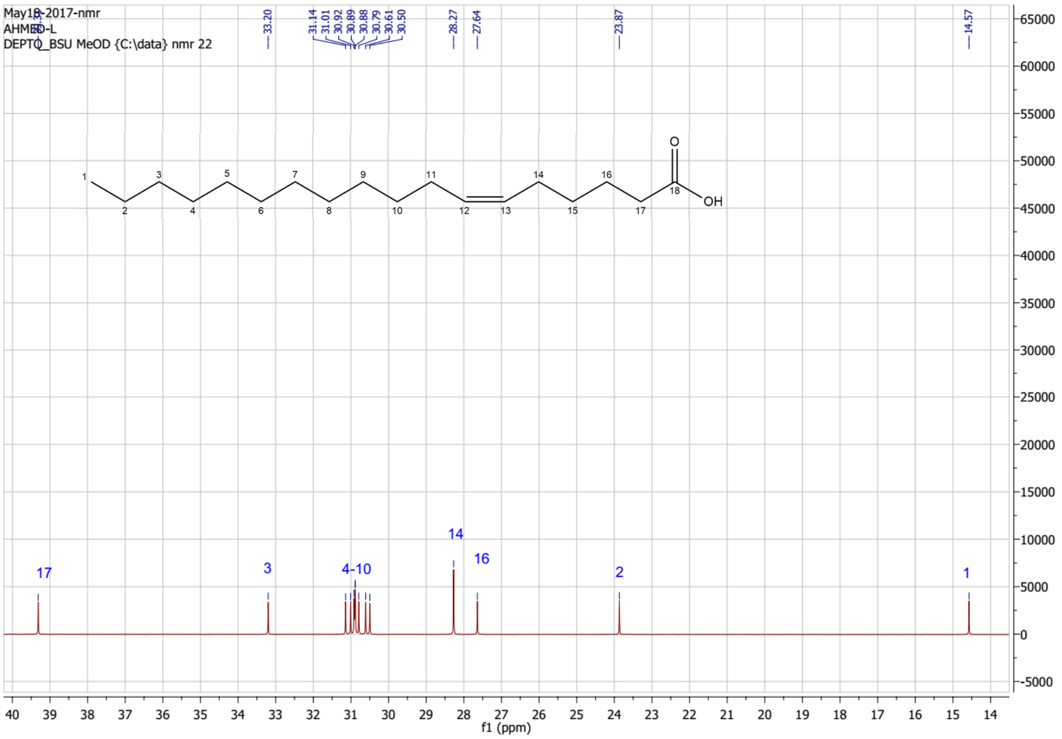

Fig.S46. ^{13}C NMR (100 MHz, $CDCl_3$) spectrum expansion of compound **10.**

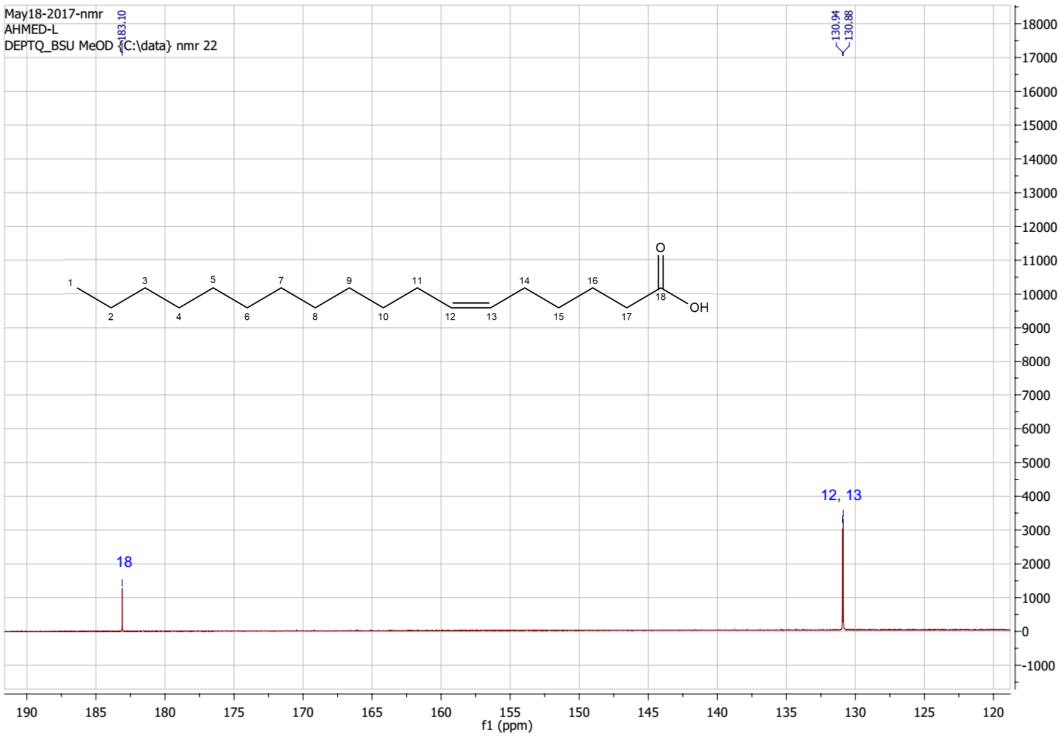

Fig.S47. ^{13}C NMR (100 MHz, $CDCl_3$) spectrum expansion of compound **10.**
